# Penicillanic Acid Sulfones Inactivate the Extended-Spectrum β-Lactamase CTX-M-15 through Formation of a Serine-Lysine Cross-Link: an Alternative Mechanism of β-Lactamase Inhibition

**DOI:** 10.1128/mbio.01793-21

**Published:** 2022-05-25

**Authors:** Philip Hinchliffe, Catherine L. Tooke, Christopher R. Bethel, Benlian Wang, Christopher Arthur, Kate J. Heesom, Stuart Shapiro, Daniela M. Schlatzer, Krisztina M. Papp-Wallace, Robert A. Bonomo, James Spencer

**Affiliations:** a School of Cellular and Molecular Medicine, University of Bristol, Bristol, United Kingdom; b Research Service, Louis Stokes Cleveland Department of Veterans Affairs, Cleveland, Ohio, USA; c Department of Proteomics and Bioinformatics, Case Western Reserve University School of Medicine, Cleveland, Ohio, USA; d School of Chemistry, University of Bristol, Bristol, United Kingdom; e Proteomics Facility, Faculty of Life Sciences, University of Bristol, Bristol, United Kingdom; f Allecra Therapeutics SAS, Saint-Louis, France; g Department of Medicine, Case Western Reserve University School of Medicine, Cleveland, Ohio, USA; h Department of Biochemistry, Case Western Reserve University School of Medicine, Cleveland, Ohio, USA; i Department of Pharmacology, Case Western Reserve University School of Medicine, Cleveland, Ohio, USA; j Department of Molecular Biology and Microbiology, Case Western Reserve University School of Medicine, Cleveland, Ohio, USA; k CWRU-Cleveland VAMC Center for Antimicrobial Resistance and Epidemiology (Case VA CARES), Cleveland, Ohio, USA; Duke University School of Medicine

**Keywords:** enmetazobactam, AAI101, tazobactam, inhibitor, antibiotic resistance, CTX-M-15, extended-spectrum β-lactamase, lysinoalanine

## Abstract

β-Lactamases hydrolyze β-lactam antibiotics and are major determinants of antibiotic resistance in Gram-negative pathogens. Enmetazobactam (formerly AAI101) and tazobactam are penicillanic acid sulfone (PAS) β-lactamase inhibitors that differ by an additional methyl group on the triazole ring of enmetazobactam, rendering it zwitterionic. In this study, ultrahigh-resolution X-ray crystal structures and mass spectrometry revealed the mechanism of PAS inhibition of CTX-M-15, an extended-spectrum β-lactamase (ESBL) globally disseminated among *Enterobacterales*. CTX-M-15 crystals grown in the presence of enmetazobactam or tazobactam revealed loss of the Ser70 hydroxyl group and formation of a lysinoalanine cross-link between Lys73 and Ser70, two residues critical for catalysis. Moreover, the residue at position 70 undergoes epimerization, resulting in formation of a d-amino acid. Cocrystallization of enmetazobactam or tazobactam with CTX-M-15 with a Glu166Gln mutant revealed the same cross-link, indicating that this modification is not dependent on Glu166-catalyzed deacylation of the PAS-acylenzyme. A cocrystal structure of enmetazobactam with CTX-M-15 with a Lys73Ala mutation indicates that epimerization can occur without cross-link formation and positions the Ser70 Cβ closer to Lys73, likely facilitating formation of the Ser70-Lys73 cross-link. A crystal structure of a tazobactam-derived imine intermediate covalently linked to Ser70, obtained after 30 min of exposure of CTX-M-15 crystals to tazobactam, supports formation of an initial acylenzyme by PAS inhibitors on reaction with CTX-M-15. These data rationalize earlier results showing CTX-M-15 deactivation by PAS inhibitors to involve loss of protein mass, and they identify a distinct mechanism of β-lactamase inhibition by these agents.

## INTRODUCTION

Antibiotic resistance among Gram-negative bacteria is of major clinical concern, due particularly to reduced efficacy of β-lactams, which account for more than half of all prescribed antibiotics (1). A major β-lactam resistance mechanism is the production of β-lactamases (2), a large family of enzymes that comprises four groups known as Ambler classes A, B, C, and D (3). Classes A, C, and D utilize an active-site serine for hydrolysis (serine-β-lactamases [SBLs]), whereas hydrolysis by class B enzymes (metallo-β-lactamases) is zinc dependent (4). CTX-M-15 (cefotaximase-Munich-15) is a class A extended-spectrum β-lactamase (ESBL) capable of hydrolyzing penicillins and cephalosporins, including third- and fourth-generation cephalosporins such as ceftazidime and cefepime, respectively (5). It is predominantly plasmid mediated, disseminated worldwide, and encountered in diverse Gram-negative pathogens, mostly *Enterobacterales*, but occasionally also in nonfermenters (6–8). CTX-M-type β-lactamases have largely supplanted SHV- and TEM-type enzymes as the most common type of ESBL (9).

CTX-M-15, like other class A SBLs, employs an acylation-deacylation mechanism to hydrolyze β-lactam antibiotics (10), utilizing four invariant residues: Ser70, Lys73, Ser130, and Glu166. Acylation occurs through attack on the β-lactam carbonyl carbon by Ser70 that has been activated by a general base (variously proposed as Glu166 or Lys73 [4, 11]). A transient tetrahedral intermediate is resolved to the acylenzyme through protonation of the amide nitrogen by Ser130, facilitated by the shuttling of protons from Glu166 to Ser130 via Lys73. The acylenzyme complex is then deacylated to release an inactive (“ring-opened”) product when a water molecule (the “deacylating water”) is activated by a general base (usually considered to be Glu166 but with possible involvement of other residues, such as Lys73 [4, 12, 13]) to attack the acylenzyme carbonyl.

**FIG 1:**
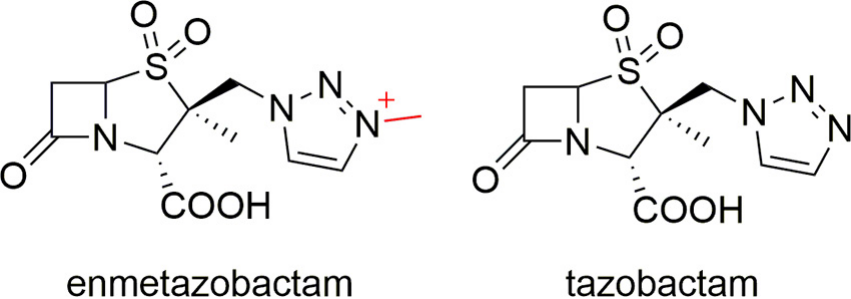
Intact enmetazobactam and tazobactam chemical structures. The additional methyl group and positive charge on the triazole ring of enmetazobactam are colored red.

Combining a β-lactam with a β-lactamase inhibitor (BLI) is a validated means of overcoming resistance to β-lactam antibiotics (14). The clinically approved “classical” BLIs clavulanic acid, sulbactam, and tazobactam all contain a β-lactam core, whereas the more recently introduced avibactam, relebactam, and vaborbactam are “nonclassical” non-β-lactam agents. All of these BLIs inhibit SBLs by forming a covalent attachment to the nucleophilic serine, with classical and some nonclassical inhibitors forming multiple breakdown products after initial acylenzyme formation (10, 14–16). However, the efficacy of BLIs has been eroded by the emergence and dissemination of β-lactamases refractory to their action (14, 17). Enmetazobactam (formerly AAI101) is a penicillanic acid sulfone (PAS) BLI that differs from tazobactam by the presence of a strategically placed methyl group on the triazole moiety (Fig. 1). This additional moiety results in a zwitterionic compound which is expected to enhance cell penetration and potency (18). The combination of enmetazobactam with the fourth-generation cephalosporin cefepime had higher *in vitro* potency than piperacillin-tazobactam against Escherichia coli strains expressing class A ESBLs, including CTX-M-15 (19–21), and was efficacious in mouse neutropenic thigh and lung infection models against ESBL-producing enterobacterial clinical isolates (19, 20, 22, 23). Compared to piperacillin-tazobactam in the ALLIUM phase 3 randomized controlled trial, cefepime-enmetazobactam demonstrated superiority at the primary endpoint in patients with complicated urinary tract infections and/or acute pyelonephritis. Superiority also was observed in the subgroup of patients infected with an ESBL-producing organism (24). The partitioning of enmetazobactam between plasma and lung epithelial lining fluid in healthy volunteers is comparable to that seen for cefepime (25).

It was determined early on that penicillanic acid sulfones inhibit β-lactamases by forming an acylenzyme complex that decomposes into multiple species, accompanied by enzyme modification (14, 21, 26–28). The modifications occur after formation of the covalent attachment to Ser70 and can also involve attack on the acylenzyme breakdown products by Ser130 to form a linked covalent bridge between Ser70 and Ser130 (28, 29). Multiple breakdown products from the initial acylenzyme can occur following reaction of PAS compounds with β-lactamases (21, 28, 30, 31). Similar intermediates also form on the reaction of the oxapenam clavulanic acid with β-lactamases (15). In this study, high-resolution crystal structures and mass spectrometry elucidated further the mechanism by which these PAS compounds inhibit CTX-M-15 (32). Our data show formation of a heretofore-unobserved direct cross-link between the two catalytically critical residues Ser70 and Lys73 that inactivates the enzyme.

## RESULTS

### Formation of a Ser70-Lys73 cross-link in CTX-M-15:PAS inhibitor cocrystal structures.

**FIG 2:**
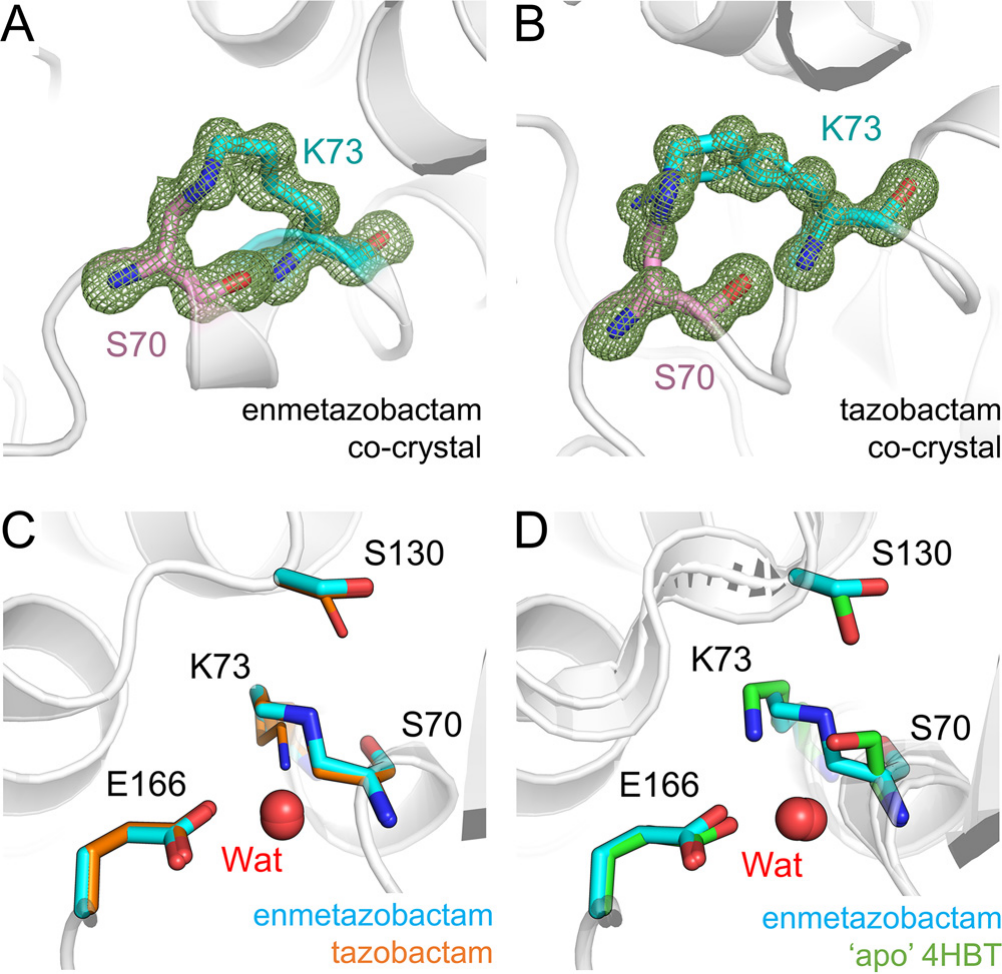
Formation of a Ser70-Lys73 lysinoalanine cross-link on PAS reaction with CTX-M-15. Views are from the active site of CTX-M-15 with protein backbones shown as gray cartoons. *F*_o_-*F*_c_ density (green mesh, contoured at 4σ) was calculated after removal of Ser70 (pink sticks) and Lys73 (cyan sticks) and clearly shows the formation of the Ser70-Lys73 cross-link. (A) CTX-M-15:enmetazobactam cocrystal structure. (B) CTX-M-15:tazobactam cocrystal structure. (C) Superposition of CTX-M-15:enmetazobactam (cyan) and tazobactam (orange) cocrystal structures (active-site residues shown as cyan sticks). The active-site water (Wat) involved in deacylation is shown as a red sphere for both structures (essentially superimposable). Dual conformations of Lys73 and Ser130 can be observed in the tazobactam crystal structure, with the minor conformers shown as thin sticks. (D) Superposition of the CTX-M-15:enmetazobactam cocrystal structure with apo CTX-M-15 (PDB code 4HBT [34], green).

Tazobactam and enmetazobactam are well established as inhibitors of CTX-M-15 *in vitro*, with 50% inhibitory concentration (IC_50_) values of 6 nM and 7 nM, respectively, against purified, recombinant enzyme, indicating very high potency (21). To elucidate further the mechanism(s) of inhibition by enmetazobactam and tazobactam, we obtained cocrystals of CTX-M-15 with each of these compounds. Crystals were harvested and cryocooled 72 h (enmetazobactam) or 3 weeks (tazobactam) after initial setup (approximately the length of time it took crystals to grow and within 24 h of their appearance) and diffracted to a resolution of 1.14 Å or 0.91 Å, respectively (Table 1). Inspection of difference maps in the active site revealed continuous electron density between Ser70 and Lys73, which was modeled as a covalent lysinoalanine cross-link with loss of the Ser70 hydroxyl group (Fig. 2A and B). This interpretation is consistent with our previous mass spectrometric data indicating a small mass reduction for CTX-M-15 following incubation with either enmetazobactam or tazobactam for up to 24 h (21). Similarly, mass spectrometric analysis of trypsin-digested protein treated with PAS inhibitors identifies peptides and ion series consistent with a Ser70-Lys73 lysinoalanine cross-link (Table 2; see also Fig. S1 in the supplemental material). Taken together with these findings, our high-resolution crystallographic data show that prolonged exposure of CTX-M-15 to either of the PAS inhibitors tazobactam and enmetazobactam results in covalent attachment of the Lys73 side chain N to the Ser70 Cβ atom and loss of the Ser70 hydroxyl.

**TABLE 1:** Crystallographic data collection and refinement statistics for CTX-M-15 cocrystals or soaks with PAS inhibitors^*a*^

Parameter	CTX-M-15:ETZ^*b*^ cross-link	CTX-M-15:TZB^*c*^ soak	CTX-M-15:TZB cross-link	CTX-M-15^E166Q^:ETZ cross-link	CTX-M-15^E166Q^:TZB cross-link	CTX-M-15^K73A^:ETZ
PDB code	6Z7J	6Z7K	7BDS	6Z7H	7BDR	7QQ5
Data collection						
Beamline	ALBA BL13-XALOC	DLS I03	Soleil PX1	ALBA BL13-XALOC	Soleil PX1	DLS I04
Space group	*P*2_1_2_1_2_1_	*P*2_1_2_1_2_1_	*P*2_1_2_1_2_1_	*P*2_1_2_1_2_1_	*P*2_1_2_1_2_1_	*P*2_1_2_1_2_1_
No. of molecules/Asymmetric unit (ASU)	1	1	1	1	1	1
Cell dimensions						
*a*, *b*, *c* (Å)	44.81, 45.99, 118.78	44.69, 45.43, 117.75	44.74, 45.58, 117.43	44.94, 45.90, 118.21	44.67, 45.67, 117.35	44.78, 45.47, 117.24
α, β, γ (°)	90.0, 90.0, 90.0	90.0, 90.0, 90.0	90.0, 90.0, 90.0	90.0, 90.0, 90.0	90.0, 90.0, 90.0	90.0, 90.0, 90.0
Wavelength (Å)	0.90	0.82	0.97856	0.90	0.97856	0.72
Resolution (Å)	45.99–1.14 (1.15–1.14)	58.88–1.10 (1.12–1.10)	58.72–0.91 (0.93–0.91)	45.90–1.42 (1.45–1.42)	45.67–0.91 (0.93–0.91)	42.39–0.95 (0.97–0.95)
*R*_pim_	0.048 (0.538)	0.027 (0.388)	0.029 (0.389)	0.059 (0.953)	0.020 (0.322)	0.050 (0.847)
CC 1/2	0.997 (0.740)	0.999 (0.787)	0.997 (0.655)	0.997 (0.427)	0.998 (0.753)	0.999 (0.357)
*I*/σ*I*	8.4 (1.3)	12.9 (1.9)	13.7 (1.9)	8.3 (1.2)	18.7 (1.9)	7.6 (0.5)
Completeness (%)	99.9 (99.2)	100.0 (100.0)	92.9 (46.5)	100.0 (100.0)	93.6 (46.7)	100.0 (99.2)
Redundancy	13.0 (12.7)	12.8 (12.2)	11.9 (5.0)	13.1 (12.8)	11.8 (4.8)	13.0 (10.8)
						
Refinement						
Resolution (Å)	41.92–1.14	58.88–1.10	58.72–0.91	42.79–1.42	42.56–0.91	42.39–0.95
No. of reflections	91,328	98,082	159,860	46,998	161,284	150,858
*R*_work_/*R*_free_	13.86/15.20	12.92/14.38	11.44/12.92	13.79/17.28	11.39/12.63	14.39/16.06
No. of non-H atoms						
Protein	2,024	2,047	2,083	2,010	2,086	2,071
Solvent	380	304	398	318	338	357
Inhibitor		20				
B-factors						
Protein	12.2	13.9	8.6	13.8	8.7	10.3
Solvent	27.7	30.4	25.0	32.1	24.3	26.2
Inhibitor		29.7				
RMS^*d*^ deviations						
Bond lengths (Å)	0.008	0.008	0.007	0.008	0.006	0.006
Bond angles (°)	1.012	1.076	1.081	1.002	1.036	0.938
Ramachandran (%)						
Outliers	0.40	0.38	0.39	0.39	0.39	0.39
Favored	98.41	98.08	98.82	98.83	98.82	98.82

a Values in parentheses are for high-resolution shell.

b ETZ, enmetazobactam.

c TZB, tazobactam.

d RMS, root mean square.

**TABLE 2:** MS/MS analysis of trypsin-digested CTX-M-15 treated with tazobactam or enmetazobactam^*a*^

Sample	Peptide	*m/z*	*M*_w_ (observed)	*M*_w_ (calculated)
Apo	FAMC^(cam)^STSK	466.2036 (2+)	931.4000	931.4012
	FAM^(ox)^C^(cam)^STSK	474.2014 (2+)	947.3956	947.3961
TZB treated	FAMC^(cam)^STSK	466.2032 (2+)	931.3992	931.4012
	**FAMCS^($)^TSK^($)^**	**428.6873 (2+)**	**856.3673**	**856.3692**
	**FAM^(ox)^CS^($)^TSK^($)^**	**436.6848 (2+)**	**872.3623**	**872.3641**
	**FAMC^(cam)^S^($)^TSK^($)^VMAAAAVLK**	**589.9706 (3+)**	**1,767.8972**	**1,767.8954**
	**FAM^(ox)^C^(cam)^S^($)^TSK^($)^VMAAAAVLK**	**595.3010 (3+)**	**1,783.8883**	**1,783.8903**
	**FAM^(ox)^C^(cam)^S^($)^TSK^($)^VM^(ox)^AAAAVLK**	**600.6324 (3+)**	**1,799.8828**	**1,799.8853**
ETZ treated	FAMC^(cam)^STSK	466.2028 (2+)	931.3982	931.4012
	FAMC^(cam)^STSKVMAAAAVLK	447.2308 (4+)	1,785.9014	1,785.9060
	**FAMCS^($)^TSK^($)^**	**428.6870 (2+)**	**856.3667**	**856.3692**
	**FAMC^(cam)^S^($)^TSK^($)^**	**457.1984 (2+)**	**913.3895**	**913.3906**
	**FAM^(ox)^C^(cam)^S^($)^TSK^($)^**	**465.1953 (2+)**	**929.3834**	**929.3855**
	**FAMC^(cam)^S^($)^TSK^($)^VMAAAAVLK**	**589.9693 (3+)**	**1,767.8933**	**1,767.8954**
	**FAM^(ox)^C^(cam)^S^($)^TSK^($)^VMAAAAVLK**	**595.3011 (3+)**	**1,783.8887**	**1,783.8903**

a Bold text indicates peptides containing a Ser70-Lys73 cross-link. $, cross-link; ox, oxidation; cam, carbamidomethylation.

10.1128/mBio.01793-21.1 FIG S1 Tandem mass spectra of tryptic peptide FAMCSTSKVMAAAAVLK of CTX-M-15. Spectra of the FAMCSTSKVMAAAAVLK peptide (residues 66 to 82, with carbamidomethylated cysteine [C^CAM^]) after treatment with tazobactam (A) or enmetazobactam (B). Several “b”- and “y” series ions were detected, but not between Ser70 and Lys73 (i.e., the cross-linked residues). A mass shift of −18 Da was observed at the precursor ion and y14, y15, and y16 ions, as well as b-series ions from b8 to b15, but not at b2 nor at y-series ions from y2 to y9, confirming formation of a lysinoalanine crosslink between Ser70 and Lys73. Download FIG S1, PDF file, 0.4 MB. Copyright © 2022 Hinchliffe et al. 2022 Hinchliffe et al. https://creativecommons.org/licenses/by/4.0/ This content is distributed under the terms of the Creative Commons Attribution 4.0 International license.

In contrast to previous crystallographic investigations of β-lactam-derived BLIs such as tazobactam or clavulanic acid (28, 30, 33), our study did not identify additional inhibitor-derived products covalently attached to Ser70. Residual positive *F*_o_-*F*_c_ density present in the active sites of both crystal structures (not covalently linked to the enzyme [Fig. S2]) could not be modeled confidently as any of the enmetazobactam/tazobactam breakdown products described previously (21) or as components of crystallization conditions, such as the SO_4_^2−^ present in the active site of apo CTX-M-15 crystallized under the same conditions. This density likely represents a mixture of different products/ligands; similar observations of poorly defined positive electron density in the active site, arising from tazobactam breakdown products, have also been noted for a cocrystal structure obtained with GES-2 (30), a class A SBL originally identified in Pseudomonas aeruginosa.

10.1128/mBio.01793-21.2 FIG S2 Residual positive *F*_o_-*F*_c_ density in the active site of CTX-M-15 cocrystallized with enmetazobactam and with tazobactam. *F*_o_-*F*_c_ density is contoured at 3σ and shown as a green mesh. The active site sulfate (labeled SO_4_^2−^) in native apo-CTX-M-15 (PDB code 4HBT [34]) is overlaid for comparison. Views are from the active sites of CTX-M-15:enmetazobactam cocrystal (green) (A) and CTX-M-15:tazobactam cocrystal (blue) (B). Download FIG S2, PDF file, 0.1 MB. Copyright © 2022 Hinchliffe et al. 2022 Hinchliffe et al. https://creativecommons.org/licenses/by/4.0/ This content is distributed under the terms of the Creative Commons Attribution 4.0 International license.

The active sites of CTX-M-15:tazobactam and CTX-M-15:enmetazobactam cocrystal structures are very similar (Fig. 2C), except that in the (ultrahigh-resolution) tazobactam cocrystal structure, Lys73, Ser130, and Asn132 could all be modeled in two conformations. In the major Ser130 conformer (occupancy, 0.6; equivalent to the conformation observed at full occupancy in the enmetazobactam cocrystal structure), the side chain is oriented away from Ser70/Lys73, an approximately 76° rotation compared to its position in the uncomplexed enzyme (PDB code 4HBT [34]). In the minor conformer (occupancy, 0.4), the Ser130 side chain points toward Lys73 (Fig. 2D). Further comparisons with uncomplexed CTX-M-15 show that cross-link formation elicits no significant change to the positioning of Glu166, the proposed general base for deacylation (4), or of the deacylating water molecule (Fig. 2D), Wat. More significantly, the newly formed alanine at position 70 resulting from loss of the Ser70 hydroxyl has epimerized, resulting in a d-amino acid with the Cα atom in the *R* configuration, which at these resolutions is defined clearly by the crystallographic data.

### Ser70 epimerization can occur without cross-link formation.

**FIG 3:**
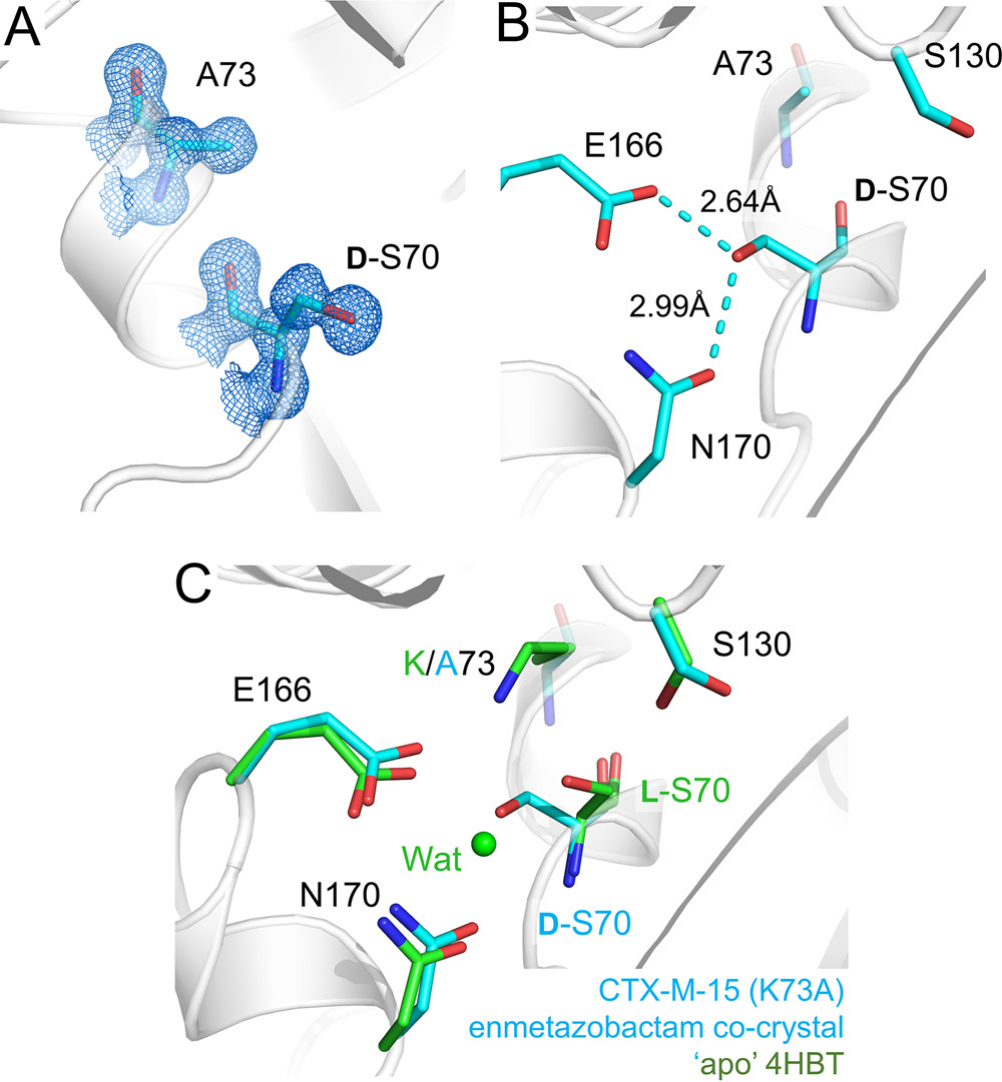
Structure of CTX-M-15^K73A^ crystallized in the presence of enmetazobactam. (A) Electron density (2*F*_o_-*F*_c_, blue, contoured at 1.2σ) of the two active-site residues Ala73 and Ser70. Enmetazobactam causes epimerization of l-Ser70 to d-Ser70, which is clearly defined by the electron density. (B) d-Ser70 is positioned to interact with active-site residue Glu166 and Asn170. (C) Overlay of CTX-M-15^K73A^ enmetazobactam cocrystal (cyan) with apo, native CTX-M-15 (PDB code 4HBT [34]). The Cα atoms of Ser70 are closely aligned, highlighting Ser70 epimerization. Note that formation of d-Ser70 results in loss of the catalytic water due to clashes with the d-Ser70 hydroxyl.

To clarify whether Ser70 epimerization results from cross-link formation, we obtained X-ray diffraction data (0.95-Å resolution) from 3-day-old cocrystals of enmetazobactam with a CTX-M-15 Lys73Ala mutant (CTX-M-15^K73A^). These data reveal l-Ser70 to have undergone epimerization to **d-**Ser70 (Fig. 3A). Comparison with the uncomplexed CTX-M-15^K73A^ structure (which at a 0.95-Å resolution [Table 3] clearly contains l-Ser70) shows that this is due to enmetazobactam exposure rather than mutation of Lys73 to Ala (Fig. S3). Epimerization leads to the d-Ser70 side chain oxygen interacting with Glu166 and Asn170 (Fig. 3B), which causes loss of the catalytic water molecule (Fig. 3C). These data show that epimerization is not dependent on cross-link formation but, rather, is a result of breakdown of the PAS-derived acylenzyme that occurs prior to formation of the Ser70-Lys73 cross-link.

**TABLE 3:** Crystallographic data collection and refinement statistics for uncomplexed CTX-M-15 mutants^*a*^

Parameter	CTX-M-15^E166Q^	CTX-M-15^K73A^	CTX-M-15^G238C^	CTX-M-15^G238C/G239_Y240insA^
PDB code	6Z7I	7QQC	7R3R	7R3Q
Data collection				
Beamline	Soleil PX2A	DLS I04	DLS I03	DLS I03
Space group	*P*2_1_2_1_2_1_	*P*2_1_2_1_2_1_	*P*2_1_2_1_2_1_	*P*2_1_2_1_2_1_
No. of molecules/ASU	1	1	1	1
Cell dimensions				
*a*, *b*, *c* (Å)	44.59, 45.69, 117.61	44.90, 45.55, 116.71	44.55, 45.65, 117.90	44.48, 45.58, 117.90
α, β, γ (°)	90.0, 90.0, 90.0	90.0, 90.0, 90.0	90.0, 90.0, 90.0	90.0, 90.0, 90.0
Wavelength (Å)	0.72	0.72	0.73379	0.73379
Resolution (Å)	45.69–0.98 (1.00–0.98)	42.43–0.95 (0.97–0.95)	58.94–1.17 (1.19–1.17)	58.95–1.456 (1.48–1.46)
*R*_pim_	0.051 (0.649)	0.043 (0.794)	0.069 (0.802)	0.097 (1.268)
CC 1/2	0.994 (0.487)	0.999 (0.368)	0.998 (0.338)	0.996 (0.419)
*I*/σ*I*	7.6 (1.1)	8.2 (0.5)	5.4 (0.5)	6.1 (0.7)
Completeness (%)	100.0 (100.0)	100.0 (99.9)	100.0 (98.6)	100.0 (100.0)
Redundancy	12.9 (12.9)	13.0 (10.7)	13.4 (13.6)	13.0 (13.5)
				
Refinement				
Resolution (Å)	42.59–0.98	35.91–0.95	58.94–1.17	58.95–1.46
No. of reflections	138,170	150,874	81,919	42,833
*R*_work_/*R*_free_	12.63/14.43	13.97/15.55	15.37/18.67	15.69/21.19
No. of non-H atoms				
Protein	2,064	2,026	2,024	2,038
Solvent	394	380	396	401
Inhibitor				
B-factors				
Protein	10.19	10.2	14.3	16.9
Solvent	29.4	24.6	29.3	30.0
Inhibitor				
RMS. deviations				
Bond lengths (Å)	0.006	0.006	0.007	0.009
Bond angles (°)	0.953	1.003	1.014	0.999
Ramachandran (%)				
Outliers	0.39	0.39	0.39	0.38
Favored	98.45	98.44	98.07	98.08

a Values in parentheses are for high-resolution shell.

10.1128/mBio.01793-21.3 FIG S3 Comparisons of CTX-M-15K73A and CTX-M-15 native structures. Views are from the active sites of CTX-M-15^K73A^:enmentazobactam (ETZ) cocrystal (cyan), apo-CTX-M-15^K73A^ (pink), and native apo-CTX-M-15 (green, PDB code 4HBT [34]). Download FIG S3, PDF file, 0.06 MB. Copyright © 2022 Hinchliffe et al. 2022 Hinchliffe et al. https://creativecommons.org/licenses/by/4.0/ This content is distributed under the terms of the Creative Commons Attribution 4.0 International license.

### Cross-link formation is not dependent on deacylation by Glu166.

To investigate the process of cross-link formation, we obtained structures from cocrystals of enmetazobactam and tazobactam with CTX-M-15^E166Q^, a conservative mutant in which Glu166 is replaced by an isosteric Gln, such that deacylation of the acylenzyme formed during β-lactam hydrolysis is impaired. Cocrystals were harvested 72 h (enmetazobactam) or 3 weeks (tazobactam) after setup (and within 24 h of their appearance, as with native enzyme) and diffracted to a resolution of 1.42 Å or 0.91 Å, respectively (Table 1). Additional diffraction data collected for uncomplexed CTX-M-15^E166Q^ (Table 3) indicate that Asn170 adopts two conformations in the E166Q-substituted active site, with the conformation observed in the native enzyme being the minor conformer (occupancy, 0.28) (Fig. S4). The major conformation (occupancy, 0.72) is also observed in a P167S/E166A double mutant of CTX-M-14 (PDB code 5VTH [35]). Gln166 in apo-CTX-M-15^E166Q^ adopts the same orientation as Glu166, with other active-site residues also in equivalent positions (Fig. S4).

10.1128/mBio.01793-21.4 FIG S4 Conformation of CTX-M-15^E166Q^ active-site residues. Superposition of unliganded CTX-M-15^E166Q^ (blue) with native, unliganded CTX-M-15 (green, PDB code 4HBT [34]). Asn170 is in two conformations, with the major conformation in CTX-M-15^E166Q^ (occupancy 0.78) oriented differently from PDB code 4HBT. The catalytic water (Wat; green or blue sphere) moves 0.8 Å due to the movement of Asn170. Mutation of Glu166 to Gln does not affect backbone or side chain geometry. Download FIG S4, PDF file, 0.05 MB. Copyright © 2022 Hinchliffe et al. 2022 Hinchliffe et al. https://creativecommons.org/licenses/by/4.0/ This content is distributed under the terms of the Creative Commons Attribution 4.0 International license.

**FIG 4:**
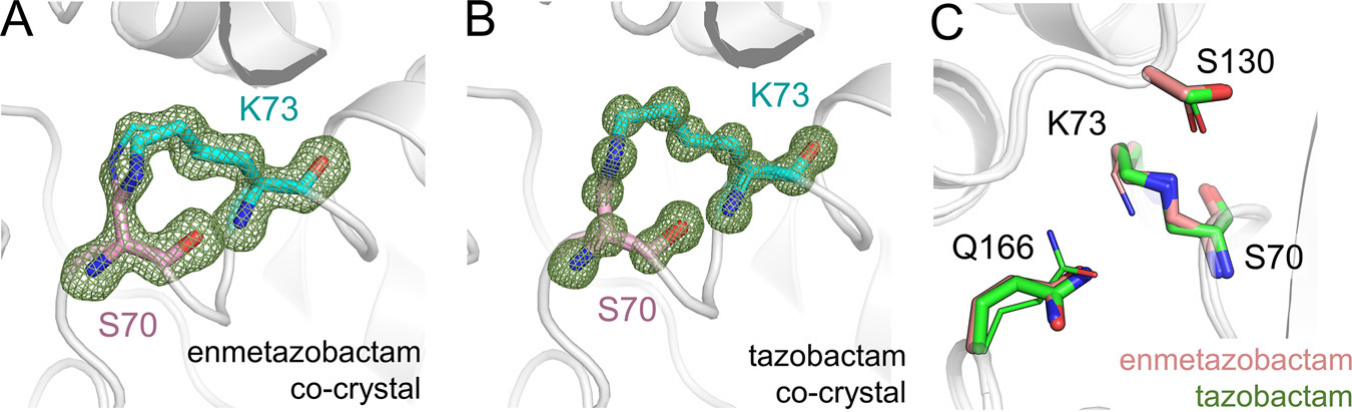
Formation of the Ser70-Lys73 lysinoalanine cross-link in cocrystal structures of enmetazobactam and tazobactam with CTX-M-15^E166Q^. Views and colors are as in Fig. 2. *F*_o_-*F*_c_ electron density is contoured at 4σ and was calculated after removal of Ser70 and Lys73 for (A) enmetazobactam and (B) tazobactam cocrystal structures. (C) Overlay of the active sites of CTX-M-15^E166Q^ cocrystallized with enmetazobactam (pink) and tazobactam (green). Note dual conformations (minor conformers shown as thin sticks) of Ser130 (both structures), Lys73 (enmetazobactam only), and Gln166 (tazobactam only).

Importantly, difference electron density maps from both the enmetazobactam and tazobactam cocrystals reveal the presence of a lysinoalanine cross-link between Ser70 and Lys73 (Fig. 4A and B). Lys73 in the CTX-M-15^E166Q^:enmetazobactam cocrystal could be modeled in two conformations (occupancies, 0.63/0.37), whereas just one conformation of Lys73 was evident in the CTX-M-15^E166Q^:tazobactam cocrystal (Fig. 4C). Conversely, Gln166 is present in two conformations (occupancies, 0.57/0.43) in the CTX-M-15^E166Q^:tazobactam cocrystal but not in the cocrystal structure with enmetazobactam. Otherwise, the two active sites are similar, with both including dual conformations of Ser130 (occupancies, 0.62/0.38 and 0.60/0.40 for tazobactam and enmetazobactam cocrystal structures, respectively). The Ser70 -Lys73 cross-link is therefore present in all four cocrystal structures presented here, with a newly formed d-alanine at position 70 in each case. Asn170 is in the same conformation in the CTX-M-15^E166Q^:enmetazobactam and CTX-M-15^E166Q^:tazobactam cocrystals as in uncomplexed wild-type CTX-M-15, with no evidence of the dual conformation observed in uncomplexed CTX-M-15^E166Q^ (Fig. S5). Asn132 in the CTX-M-15^E166Q^:tazobactam cocrystal structure could be modeled in three conformations (occupancies, 0.49/0.27/0.24), possibly as a result of the dual conformation of Gln166 but also reflecting the extremely high resolution of this structure, allowing detection of all three conformations (Fig. S5B). Despite these minor differences, however, the structures clearly demonstrate that cross-link formation is independent of Glu166-mediated deacylation.

10.1128/mBio.01793-21.5 FIG S5 Movements of active-site residues in CTX-M-15^E166Q^:PAS cocrystal structures. (A) Superposition of CTX-M-15^E166Q^:enmetazobactam (pink) with native, unliganded, CTX-M-15 (green, PDB code 4HBT [34]). Asn170 adopts one conformation, identical to that in PDB code 4HBT. However, there is no electron density for the catalytic water in CTX-M-15^E166Q^:enmetazobactam. (B) Superposition of CTX-M-15^E166Q^:tazobactam (pink) cocrystal structure with unliganded CTX-M-15^E166Q^ (blue). Download FIG S5, PDF file, 0.07 MB. Copyright © 2022 Hinchliffe et al. 2022 Hinchliffe et al. https://creativecommons.org/licenses/by/4.0/ This content is distributed under the terms of the Creative Commons Attribution 4.0 International license.

As noted above, previous mass spectrometry experiments (21) established that extended exposure of wild-type CTX-M-15 to PAS compounds results in loss of protein mass. The possibility that CTX-M-15^E166Q^ exhibits similar behavior was investigated. A corresponding mass loss was confirmed by electrospray mass spectrometry of CTX-M-15^E166Q^ after incubation with tazobactam or enmetazobactam (Fig. S6). These data indicate that both PAS BLIs can rapidly (within 1 min) react with CTX-M-15^E166Q^, as evidenced by the presence of multiple PAS-derived covalent species, identified as +91-Da, +69-Da, and +50-Da adducts (peaks 1, 2, and 3 in Fig. S6A). Formation of the Ser70-Lys73 cross-link, identified as an 18-Da mass loss, appeared after 15 min and represents the primary species present after a 24-h incubation. Peaks corresponding to covalently linked PAS breakdown products were no longer evident at this time point. Enzyme activity assays revealed low-level hydrolysis of the chromogenic cephalosporin nitrocefin by CTX-M-15^E166Q^ (indicating that antibiotic turnover by this mutant is impaired) which was abolished after incubation with PAS compounds for 24 h (Fig. S6B). Analysis of trypsin-digested protein also revealed cross-link formation (Table 4 and Fig. S7). Therefore, as previously observed for wild-type CTX-M-15, the Ser70-Lys73 lysinoalanine cross-link can be formed by, and inactivate, CTX-M-15^E166Q^ (21), which also retains the ability to be acylated by, and subsequently deacylate complexes of, PAS BLIs.

**TABLE 4:** MS/MS analysis of trypsin-digested CTX-M-15^E166Q^ treated with tazobactam or enmetazobactam^*a*^

Sample	Peptide	*m/z*	*M*_w_ (observed)	*M*_w_ (calculated)
Apo	FAMCSTSK	437.6934 (2+)	874.3794	874.3797
	FAM^(ox)^CSTSK	445.6902 (2+)	890.3732	890.3747
	FAMC^(cam)^STSK	466.2037 (2+)	931.4001	931.4012
	FAM^(ox)^C^(cam)^STSK	474.2013 (2+)	947.3953	947.3961
TZB treated	FAMCSTSK	437.6928 (2+)	874.3783	874.3797
	FAMC^(cam)^STSK	466.2039 (2+)	931.4005	931.4012
	FAMC^(cam)^STSKVMAAAAVLK	447.2319 (4+)	1,785.9059	1,785.9060
	FAM^(ox)^C^(cam)^STSKVMAAAAVLK	601.3008 (3+)	1,801.8878	1,801.9009
	**FAMCS**^($)^**TSK**^($)^****	**428.6876 (2+)**	**856.3678**	**856.3692**
	**FAM^(ox)^CS($)TSK^($)^**	**436.6857 (2+)**	**872.3641**	**872.3641**
	**FAMC^(cam)^S^($)^TSK^($)^**	**457.1988 (2+)**	**913.3904**	**913.3906**
	**FAMC^(cam)^S^($)^TSK^($)^VMAAAAVLK**	**589.9701 (3+)**	**1,767.8957**	**1,767.8954**
	**FAM^(ox)^C^(cam)^S^($)^TSK^($)^VMAAAAVLK**	**595.3016 (3+)**	**1,783.8904**	**1,783.8903**
	**FAM^(ox)^C^(cam)^S^($)^TSK^($)^VM^(ox)^AAAAVLK**	**600.6336 (3+)**	**1,799.8863**	**1,799.8853**
ETZ treated	FAMCSTSK	437.6935 (2+)	874.3797	874.3797
	FAMC^(cam)^STSK	466.2035 (2+)	931.3998	931.4012
	FAMC^(cam)^STSKVMAAAAVLK	447.2319 (4+)	1,785.9059	1,785.9060
	FAM^(ox)^C^(cam)^STSKVMAAAAVLK	601.3005 (3+)	1,801.8871	1,801.9009
	**FAMCS^($)^TSK^($)^**	**428.6879 (2+)**	**856.3685**	**856.3692**
	**FAM^(ox)^CS^($)^TSK^($)^**	**436.6858 (2+)**	**872.3643**	**872.3641**
	**FAMC^(cam)^S^($)^TSK^($)^**	**457.1991 (2+)**	**913.3910**	**913.3906**
	**FAMC^(cam)^S^($)^TSK^($)^VMAAAAVLK**	**589.9702 (3+)**	**1,767.8959**	**1,767.8954**
	**FAM^(ox)^C^(cam)^S^($)^TSK^($)^VMAAAAVLK**	**595.3019 (3+)**	**1,783.8911**	**1,783.8903**
	**FAM^(ox)^C^(cam)^S^($)^TSK^($)^VM^(ox)^AAAAVLK**	**600.6330 (3+)**	**1,799.8844**	**1,799.8853**

a Bold text indicates peptides containing a Ser70-Lys73 cross-link.

10.1128/mBio.01793-21.6 FIG S6 Intermediates formed on reaction of PAS compounds with CTX-M-15^E166Q^ revealed by timed electrospray ionization-mass spectrometry (ESI-MS). (A) Mass spectra showing the formation of intermediates during inactivation of CTX-M-15^E166Q^ (apo; 28,257 Da) by enmetazobactam or tazobactam at 1-min, 15-min, and 24-h incubations. Previous work conducted by Papp-Wallace et al. (21) (Fig. 4 in main text) showed the ESI-MS of apo-CTX-M-15 and complexed with enmetazobactam and tazobactam. As determined by analyzing ESI-MS of CTX-M-15^E166Q^, the deacylation-deficient variant peaks 1 (+90 Da), 2 (+69 Da), and 3 (+50 Da) correspond to postacylation fragmentation of the two PAS compounds from the enamine/imine intermediates (Fig. 6 in main text; see also reference 21). Peak 4 is the −18-Da modified apoenzyme (containing a Ser70-Lys73 lysinoalanine cross-link Fig. 7 in main text). (B) Possible breakdown products corresponding to peaks 1, 2, and 3 in panel A, as identified previously (21). Two breakdown products of equivalent mass, including the Ser70-Ser130 cross-linked species (21, 28, 29) may be represented by peak 3. Adducts at ~50 Da represented by peak 3 have also been shown to form on reaction of PAS compounds with both GES-2 (34) and KPC-2 (21) and can break down to yield active enzyme (KPC-2) or, as in this study, lead ultimately to formation of the catalytically inactive Ser70-Lys73 cross-linked species. (C) Nitrocefin hydrolysis by CTX-M-15^E166Q^ after 24 h of incubation with enmetazobactam (red) or tazobactam (blue). The observed hydrolysis by the CTX-M-15^E166Q^:enmetazobactam protein after 24 h of incubation is due to the small amount of unmodified enzyme present in this mixture (see mass spectrum at 24-h timepoint, panel A, bottom left). Download FIG S6, PDF file, 0.4 MB. Copyright © 2022 Hinchliffe et al. 2022 Hinchliffe et al. https://creativecommons.org/licenses/by/4.0/ This content is distributed under the terms of the Creative Commons Attribution 4.0 International license.

10.1128/mBio.01793-21.7 FIG S7 Tandem mass spectra of tryptic peptide FAMCSTSKVMAAAAVLK of CTX-M-15^E166Q^. Spectra of the FAMCSTSKVMAAAAVLK peptide (residues 66 to 82, with carbamidomethylated cysteine) after treatment with tazobactam (A) or enmetazobactam (B). Several b- and y-series ions were detected but not between Ser70 and Lys73 (i.e., the cross-linked residues). A mass shift of −18 Da was observed at the precursor ion and y14, y15, and y16 ions, as well as b-series ions from b8 to b15, but not at b2 nor at y-series ions from y2 to y9, confirming formation of a lysinoalanine crosslink between Ser70 and Lys73. Download FIG S7, PDF file, 0.2 MB. Copyright © 2022 Hinchliffe et al. 2022 Hinchliffe et al. https://creativecommons.org/licenses/by/4.0/ This content is distributed under the terms of the Creative Commons Attribution 4.0 International license.

### Cross-link formation is retained in the presence of an active-site disulfide.

Our previous study (21) identified a loss in mass consistent with cross-link formation after exposure of the SHV-1 and CTX-M-15 β-lactamases to tazobactam or enmetazobactam but not with the carbapenemase KPC-2. One possible explanation for this is the presence in KPC-2 of a Cys69-Cys238 disulfide bond, a distinguishing feature of carbapenem-hydrolyzing class A enzymes (4, 36). To test whether a disulfide could prevent cross-link formation, we constructed a CTX-M-15 Gly238Cys mutation (CTX-M-15^G238C^) which already contains a Cys residue at position 69 and used mass spectrometry to investigate whether this mutant could support cross-link formation. As carbapenem-hydrolyzing class A β-lactamases also contain a single amino acid insertion at position 240 (compared to the equivalent region in ESBLs such as CTX-M-15), we constructed an additional variant containing an alanine insertion at position 240 (CTX-M-15^G238C/G239_Y240insA^) to more closely resemble the equivalent loop in class A carbapenemases (37–40, 56). Formation of a Cys69-Cys238 disulfide was confirmed by determining the crystal structures of both mutants (Table 3 and Fig. S8). As shown in Fig. S8, neither mutant prevented cross-link formation, as evidenced by the presence of a peak corresponding to a mass loss of 18-Da on 24 h of exposure to tazobactam or enmetazobactam. Thus, presence of an active-site disulfide bond is not sufficient to abolish cross-link formation by CTX-M-15 and does not account for the absence of cross-link formation in KPC-2.

10.1128/mBio.01793-21.8 FIG S8 Effect of a C 69–C 238 disulfide bridge on cross-link formation in CTX-M-15. Shown is disulfide formation in the CTX-M-15 mutants defined by 2*F*_o_-*F*_c_ electron density (blue mesh, contoured at 1σ): CTX-M-15^G238C^ (A) and CTX-M-15^G238C/G239_Y240insA^ (B). (C) Overlay of CTX-M-15^G238C^ (cyan), CTX-M-15^G238C/G239_Y240insA^ (green), and KPC-2 PDB code 5UL8 [56], magenta active sites. Note that the CTX-M-15^G238C/G239_Y240insA^ loop is structurally similar to the equivalent loop in the KPC-2 carbapenemase, in which cross-link formation following PAS exposure is not observed (21). (D) and (E) Mass spectra, at bottom, showing the formation of intermediates during inactivation of CTX-M-15 mutants by enmetazobactam or tazobactam after 24-h incubation for CTX-M-15^G238C^ (apo, 28,306 Da) (D) and CTX-M-15^G238C/G239_Y240insA^ (apo, 28,377 Da) (E). Peak 4 is the −18-Da modified apo enzyme representing formation of the Ser70-Lys73 lysinoalanine cross-link. Download FIG S8, PDF file, 0.5 MB. Copyright © 2022 Hinchliffe et al. 2022 Hinchliffe et al. https://creativecommons.org/licenses/by/4.0/ This content is distributed under the terms of the Creative Commons Attribution 4.0 International license.

### Tazobactam forms an imine acylenzyme with CTX-M-15.

**FIG 5:**
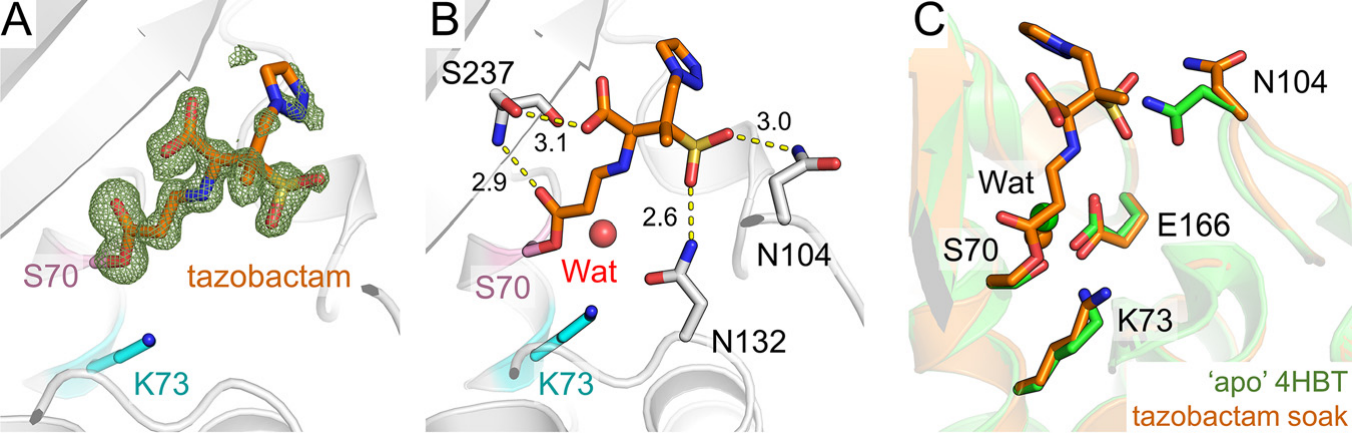
Crystal structure of tazobactam bound to CTX-M-15. The CTX-M-15 backbone is colored gray, tazobactam is colored orange, and Ser70 and Lys73 are shown as pink and gray sticks, respectively. Tazobactam is shown as orange sticks. (A) *F*_o_-*F*_c_ electron density (green, 3σ) was calculated after removal of tazobactam from the model. (B) Interactions (yellow dashes) of tazobactam with residues (gray sticks) in the CTX-M-15 active site are shown with distances labeled in angstroms. (C) Active site of unliganded apo CTX-M-15 (green, PDB code 4HBT [34]) overlaid with CTX-M-15:tazobactam (orange). The active-site catalytic waters are shown as spheres colored according to protein.

We next sought to determine the mode of CTX-M-15 inhibition by PAS compounds at time points prior to cross-link formation. To this end, crystals of uncomplexed wild-type CTX-M-15 were soaked in enmetazobactam or tazobactam for periods ranging from 1 min to 24 h before being snap-frozen for diffraction data collection. While the majority of these experiments yielded no electron density suggestive either of an intact PAS ligand in the active site or of PAS fragmentation products, we were able to determine a structure of CTX-M-15 with bound tazobactam from a crystal soaked in inhibitor for 30 min. Diffraction data extended to 1.1-Å resolution (Table 1), with the *F*_o_-*F*_c_ density indicating covalent attachment of a hydrolyzed form of tazobactam to Ser70 (Fig. 5A). Electron density was well resolved for all of the tazobactam-derived product excepting the triazole moiety, which does not interact with the protein main chain and is likely to be mobile, resulting in higher crystallographic B-factors over these atoms (average, 43.0 Å^2^ versus 29.7 Å^2^ over all inhibitor atoms). In this structure, the carbonyl oxygen of the covalently attached tazobactam-derived product interacts with the backbone N of Ser237 in the oxyanion hole, while the sulfone oxygen interacts with the nitrogen atom of the Asn132 side chain (Fig. 5B). Binding also results in disruption of residues 103 to 106, resulting in B-factors that are 2.9-fold greater than the protein average (over all atoms), which appears necessary to avoid a steric clash between tazobactam and Asn104 (Fig. 5C).

**FIG 6:**
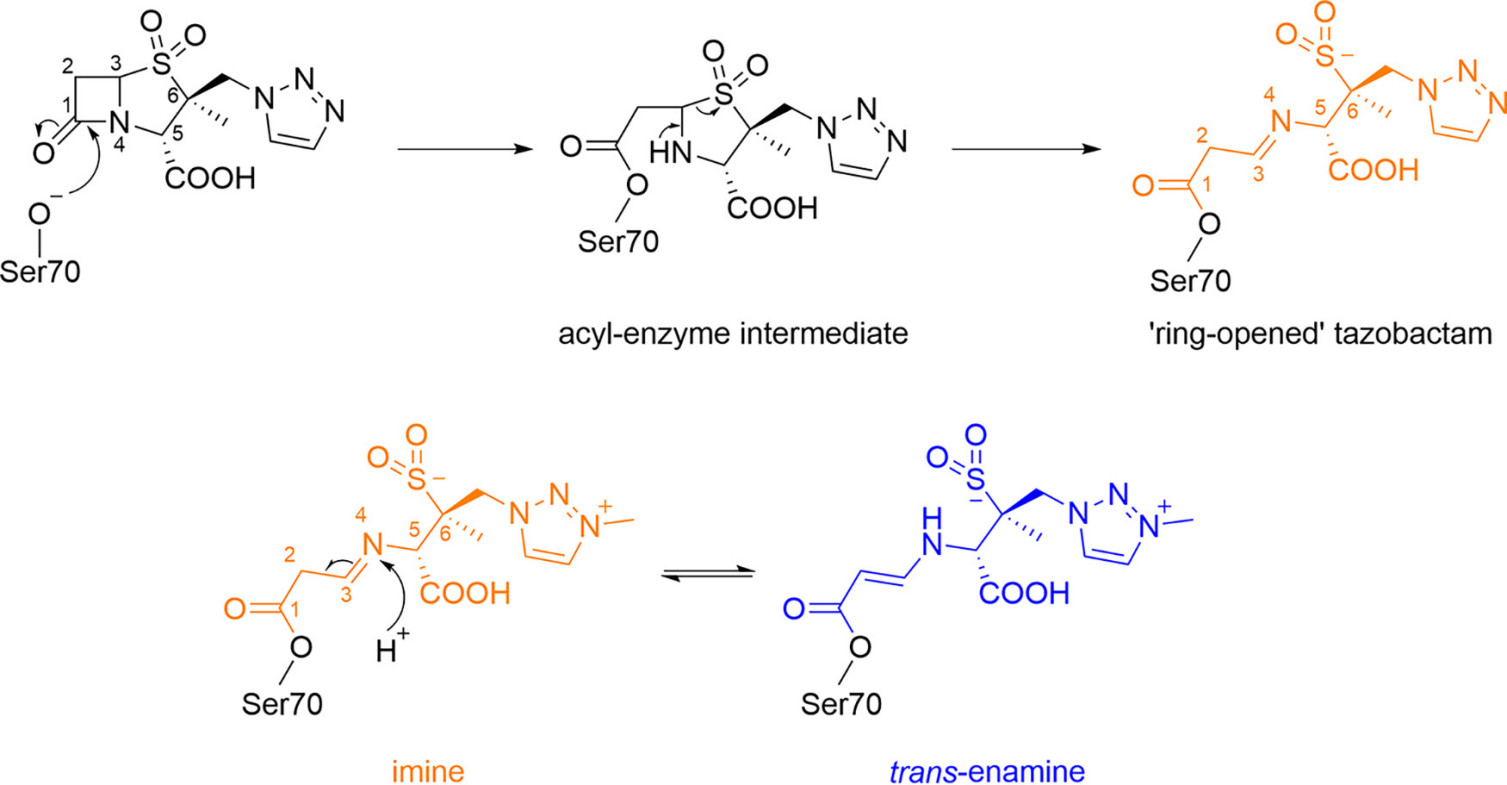
General mechanism for tazobactam/enmetazobactam acylenzyme formation and rearrangement by class A β-lactamases. (Top) Schematic of tazobactam acylenzyme formation and ring opening on reaction with serine-β-lactamases. The same reaction sequence applies to enmetazobactam. *Bottom*, The imine form left, orange, as modeled in our crystal structure (Fig. 5) of PAS inhibitors (enmetazobactam shown) can convert to the *trans*-enamine form (right, blue) via protonation at N4 and tautomerization of the double bond. See Fig. 5 for interactions of the imine form with CTX-M-15. Fragmentation of the imine/enamine leads to the degradation products observed by mass spectrometry (21) (Fig. S6).

Tazobactam forms multiple breakdown products on binding to class A β-lactamases (21, 30, 31), with initial hydrolysis and ring opening (Fig. 6) resulting in formation of *cis*-enamine, imine, or *trans*-enamine products. The crystal structure obtained in this study indicates a clearly linear molecule that is either the imine or *trans*-enamine product of tazobactam (Fig. 6). We modeled the imine form based on our previous molecular dynamics simulations that identified the imine form as the more energetically favorable in CTX-M-15 (21). This assignment is supported by the high quality of our experimental electron density maps (1.1-Å resolution, coordinate error of ±0.09 Å) which are suggestive of the imine form of tazobactam, as evidenced by the location of difference peaks consistent with the expected atom locations for a C = N bond (Fig. S9). However, despite the high quality of our data, we cannot rule out the possibility that the captured structure contains either the *trans*-enamine intermediate or a mixture/equilibrium of the imine/*trans*-enamine forms. The stability of different PAS-derived intermediates in the β-lactamase active site is likely enzyme and environment specific; Raman spectroscopy has shown the imine form to be the most stable in OXA-10 (41) and the *trans*-enamine more stable in SHV-1 (16), while in CTX-M-9 these intermediates can be readily attacked by Ser130 to form a β-alkoxyacrylate via a Ser70-Ser130 covalent bridge (42).

10.1128/mBio.01793-21.9 FIG S9 Omit electron density for tazobactam bound to CTX-M-15. *F*_o_-*F*_c_ density calculated in the absence of ligand is contoured at (A) 8σ or (B) 9σ. At this resolution (1.1 Å), the C = N bond length observed in the crystal structure (1.29 Å) is shorter than the expected values (F. H. Allen, O. Kennard, D. G. Watson, L. Brammer, et al., Perkin transactions 2:S1–S19, 1987, https://doi.org/10.1039/P298700000S1) for a C-N or C-C bond (1.47 Å/1.50 Å, respectively). Download FIG S9, PDF file, 0.1 MB. Copyright © 2022 Hinchliffe et al. 2022 Hinchliffe et al. https://creativecommons.org/licenses/by/4.0/ This content is distributed under the terms of the Creative Commons Attribution 4.0 International license.

In a previously determined structure of tazobactam bound to the class A β-lactamase GES-2 (PDB code 3NIA [30]), the ring-opened PAS was covalently bound to Ser70, but at 1.65-Å resolution it was not possible for the authors to state definitively whether the imine or *trans*-enamine breakdown product was observed. Other crystal structures in which hydrolyzed tazobactam has been modeled into native class A β-lactamases, such as BlaC (PDB code 6H2I, 2.75-Å resolution [33]), BS3 (4A5R, 2.1-Å resolution [unpublished data]), and SHV-1 1VM1, 2.02-Å resolution [28], are of too low a resolution and/or feature insufficiently defined inhibitor-derived acylenzyme for definitive assignment of the tautomeric state. In the highest-resolution tazobactam complex structure previously available (GES-2, PDB code 3NIA [30] [Fig. S10]), the inhibitor-derived product binds in an orientation almost identical to that seen in CTX-M-15, except that in the GES-2 complex the flexible triazole ring is flipped by a rotation at C-6. However, the tazobactam-derived product makes only one interaction (with Thr237 in the oxyanion hole) with the protein main chain of GES-2, compared to multiple interactions in CTX-M-15. This may account for the nearly 100-fold difference in IC_50_ values observed for tazobactam inhibition of GES-2 (500 nM [43]) and CTX-M-15 (7 nM [21]).

10.1128/mBio.01793-21.10 FIG S10 Comparison of tazobactam binding to GES-2 and CTX-M-15. Shown is a superposition of CTX-M-15:tazobactam (orange, 1.1-Å resolution) with GES-2:tazobactam [blue, PDB code 3NIA [30], 1.65 Å resolution]. Interactions are shown as colored dashes corresponding to the protein color. In GES-2:tazobactam, the ring-opened form of tazobactam interacts only with the backbone amide of Ser237. Download FIG S10, PDF file, 0.06 MB. Copyright © 2022 Hinchliffe et al. 2022 Hinchliffe et al. https://creativecommons.org/licenses/by/4.0/ This content is distributed under the terms of the Creative Commons Attribution 4.0 International license.

## DISCUSSION

Enmetazobactam is a penicillanic acid sulfone that is a potent inhibitor of ESBLs such as CTX-M-15 (21). Based on X-ray crystallography, we now describe a novel mode of β-lactamase inactivation by enmetazobactam and tazobactam, involving formation of a lysinoalanine cross-link between Ser70 and Lys73, two catalytically important residues in the active sites of class A β-lactamases, including CTX-M-15.

The crystallographic data presented herein complement our mass spectrometric observations that enmetazobactam and tazobactam behave similarly toward CTX-M-15, with the same mass loss evident at similar time points after exposure (21). Differences in their effects on β-lactam MICs toward β-lactamase-producing Gram-negative bacteria, evidenced by an 8-fold-lower MIC_90_ of cefepime-enmetazobactam (1 μg/mL) relative to cefepime-tazobactam (8 μg/mL) against ESBL-producing Klebsiella pneumoniae (23), may be attributable to differences in their respective abilities to access the periplasm, reflecting the zwitterionic nature of enmetazobactam. The extent of cross-link formation in the bacterial cell remains uncertain, although given that this can be observed after 15 min of incubation *in vitro*, it may contribute to inhibition over the time course of an MIC assay.

Initial interaction of PAS compounds with CTX-M-15 generates an acylenzyme which decomposes into multiple products, accompanied by formation of the Ser70-Lys73 lysinoalanine cross-link (seen as a reduction in protein mass) that slowly accumulates over time, leading to a fully inactivated (i.e., fully cross-linked) enzyme after a 24-h incubation. The CTX-M-15:tazobactam complex structure, obtained after only a 30-min incubation of crystal with inhibitor, shows how PAS compounds may form the initial acylenzyme (Fig. 5). To date, this is the highest-resolution structure of tazobactam bound to any β-lactamase, with the electron density indicative of the ring-opened imine intermediate. However, we cannot fully rule out the presence of the *trans*-enamine intermediate, with the possibility that the two forms tautomerize. As previously noted for GES-2, interactions with the β-lactamase active site do not appear to exert any steric constraints preventing formation of one or the other tautomer (30), although it is possible that conversion from the imine to the *trans*-enamine is slower *in crystallo* than in solution, effectively trapping the imine form in our crystal structure.

Our crystallographic and mass spectrometry data show that Glu166 is not required for deacylation or cross-link formation by either tazobactam or enmetazobactam. The E166Q mutant also does not affect avibactam decarbamylation by CTX-M-15 (44), in which neutral Lys73 accepts a proton from Ser130 to initiate intramolecular recyclization and removal of the covalently attached diazabicyclooctane inhibitor. However, as recyclization does not occur with PAS inhibitors, it is unlikely that the same mechanism drives PAS deacylation and cross-link formation. It remains to be seen whether an active-site water molecule or other catalytic residues, such as Ser130, are involved in deacylation of PAS-derived acylenzyme species. However, with no interactions identifiable between Ser130 and the covalently attached tazobactam-derived product, and with Ser130 oriented away from the cross-link in our cocrystal structures (Fig. 2), the current data do not support involvement of Ser130 in formation of the Ser70-Lys73 cross-link.

**FIG 7:**
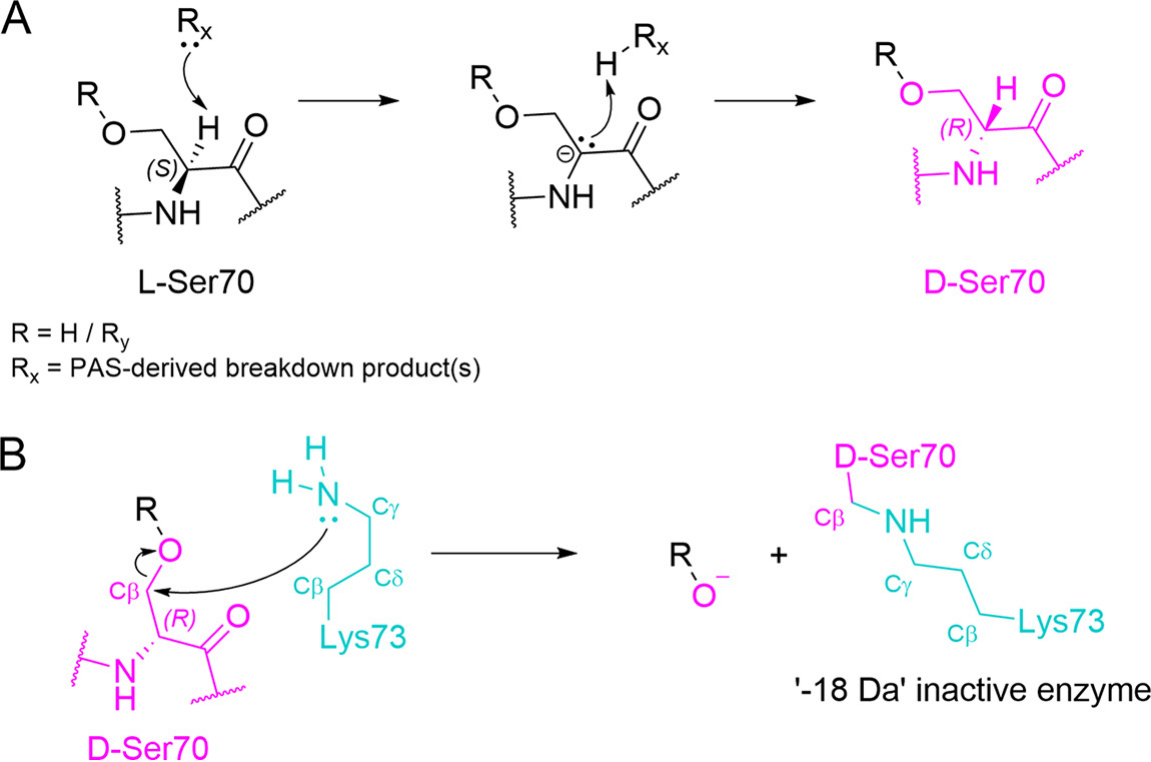
Possible route of Ser70-Lys73 cross-link formation in CTX-M-15. (A) Epimerization of acylated l-Ser70 to d-Ser70 (pink) involving abstraction of the Cα hydrogen by a fragment of PAS-derived product released on PAS breakdown (R_x_, where R_x_ may refer to one or more different breakdown products). R could be a hydrogen (i.e., regenerated Ser70, as seen in the CTX-M-15^K73A^ structure) or R_y_ (representing a PAS-derived product[s]) that remains once R_x_ leaves. Note that the strong base R_x_ remains to be identified (B) Direct attack on d-Ser70 (pink) Cβ by the Lys73 (cyan) N results in addition of the Ser70 O to the PAS-derived product, loss of the inhibitor-derived leaving group (R), and subsequent formation of the Ser70-Lys73 cross-link.

**FIG 8:**
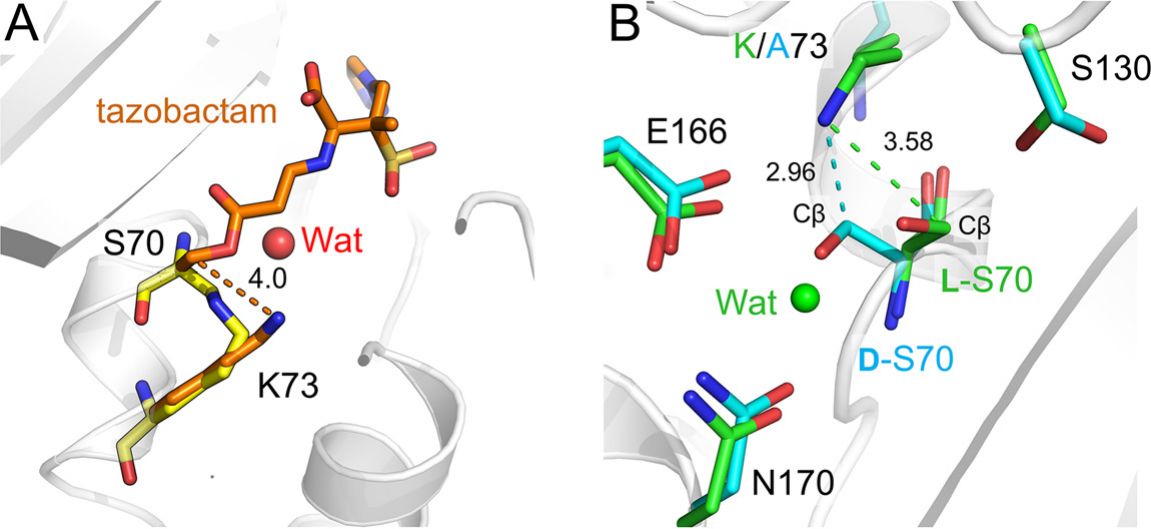
Distances between Lys73 and the Ser70 Cβ. View from the active sites, with distances between Lys73 Nζ and the Cβ of Ser70 labeled in angstroms. (A) CTX-M-15:enmetazobactam cocrystal (yellow) overlaid with native CTX-M-15:tazobactam-derived acylenzyme (orange). (B) CTX-M-15^K73A^:enmetazobactam cocrystal (cyan) overlaid with uncomplexed CTX-M-15 (PDB code 4HBT [34]).

A noteworthy feature of four of the PAS cocrystal structures presented here is the presence of a d-alanyl residue at position 70. For this to be generated, the Cα atom of Ser70 must be deprotonated by a strong base arising as one or more breakdown products formed after reaction of the PAS compounds with CTX-M-15 (21) (Fig. 7A). Identifying the exact chemical structure(s) of the strong base(s) responsible requires further investigation. Previous studies have shown that the protonation state of Lys73 in CTX-M β-lactamases can be affected by inhibitor binding but that this residue is usually electrostatically neutral (12, 45). If Lys73 directly initiates cross-link formation, this may occur via a neutral lysine (activated by PAS binding or by PAS breakdown products in the active site) attacking the Cβ atom of d-Ser70, causing inhibitor deacylation, removal of the Ser70 side chain oxygen, and formation of a Ser70 -Lys73 lysinoalanine cross-link (Fig. 7B). Epimerization is likely required to facilitate this attack, as in the CTX-M-15:tazobactam structure Lys73 Nζ is situated 4.0 Å from the Cβ of l-Ser70, necessitating movement to initiate cross-link formation (Fig. 8A). Indeed, when the CTX-M-15^K73A^:enmetazobactam cocrystal structure, in which Ser70 has epimerized but the cross-link has not formed, is superimposed upon the structure of uncomplexed CTX-M-15, the d-Ser70 Cβ is 2.9 Å from the Lys73 Nζ (Fig. 8B). The identity of the covalently linked fragmentation product (R group in Fig. 7) present at the time of cross-link formation is unknown, despite our extensive efforts aimed at capturing bound products by cryocooling CTX-M-15 crystals exposed to PAS compounds at different time points during the reaction. The existence of multiple fragmentation pathways for PAS BLIs (16, 21) makes it likely that more than one species may support cross-link formation, making identification of the Ser70-bound degradation product immediately preceding cross-link formation especially difficult. Therefore, we cannot rule out involvement in cross-link formation of intermediates yet to be identified, including those involved in Ser70 epimerization.

Our previous study (21) found that the outcome of reaction with PAS compounds differs between different class A β-lactamases. Specifically, mass spectrometry provided no evidence for cross-link formation by KPC-2. Carbapenemases such as KPC-2 differ from most other class A enzymes by possessing a Cys69-Cys238 disulfide bond in the active site, which is important for their stability (4, 36, 40, 46) but by limiting active-site conformational flexibility may constrain the orientation of bound substrates. As a further investigation of the importance of active-site movement to cross-link formation, we sought to constrain the CTX-M-15 active site through introduction of the equivalent disulfide by directed mutagenesis. This, however, did not affect the ability of mutant enzymes CTX-M-15^G238C^ and CTX-M-15^G238C/G239_Y240insA^ to form cross-links, indicating that differences in disulfide content (and possibly a concurrent reduction in active site flexibility) are not the explanation for the differing abilities of CTX-M-15 and KPC-2 to support cross-link formation.

The crystallographic data presented here rationalize our earlier biochemical results by identifying the time-dependent mass loss on incubation of CTX-M-15 with penicillanic acid sulfones as corresponding to formation of an unprecedented Ser70-Lys73 lysinoalanine cross-link, with concomitant epimerization of Ser70. These findings expand the range of mechanisms by which serine β-lactamases can be inactivated by β-lactam-derived inhibitors.

## MATERIALS AND METHODS

### Purification and crystallization of CTX-M-15.

CTX-M-15 was expressed, purified, and crystallized as described previously (10). CTX-M-15 mutants were generated with the primer pairs 5′-GTCTCGACCGTACCCAGCCGACGTTAAAC-3′ and 5′-GTTTAACGTCGGCTGGGTACGGTCGAGAC-3′ (CTX-M-15^E166Q^), 5′-GCGGCCATCACTGCACTGGTGCTGCACATCGCAAAG-3′ and 5′-CTTTGCGATGTGCAGCACCAGTGCAGTGATGGCCGC-3′ (CTX-M-15^K73A^), and 5′-GATAAAACCGGCAGCTGTGGCTATGGCACCAC-3′ and 5′-GTGGTGCCATAGCCACAGCTGCCGGTTTTATC-3′ (CTX-M-15^G238C^) using a QuikChange Lightning site-directed mutagenesis kit (Agilent Technologies, Inc, Santa Clara, CA) according to manufacturer’s instructions and the native popinF-CTX-M-15 (47) as the template. CTX-M-15^G238C/G239_Y240insA^ was generated using popinF-CTX-M-15^G238C^ as a template with the primer pair 5′-GATAAAACCGGCAGCTGTGGCGCGTATGGCACCACCAAC-3′ and 5′-GTTGGTGGTGCCATACGCGCCACAGCTGCCGGTTTTATC-3′. All mutant enzymes were purified using the same procedure as for the native enzyme and crystallized using seeds generated from native CTX-M-15 crystals (10), as previously described (48).

For cocrystallization experiments with enmetazobactam (Allecra Therapeutics SAS, Saint-Louis, France) and tazobactam (Sigma Chemical Corp., St. Louis, MO), 2 μL of purified protein (CTX-M-15, CTX-M-15^E166Q^, or CTX-M-15^K73A^) was added to 2 μL of 10 mM PAS [prepared in CTX-M-15 mother liquor: 0.1 M Tris (pH 8.0) and 2.0 to 2.4 M (NH_4_)_2_SO_4_] and equilibrated against 500 μL of crystallization reagent in CrysChem 24-well plates (Hampton Research Corp., Aliso Viejo, CA) at 19°C. For all mutants, cocrystals grew within 2 days (enmetazobactam) or 3 weeks (tazobactam).

### Inhibitor soaking, data collection, and structure determination.

CTX-M-15 crystals were soaked in mother liquor supplemented with 2.5 mM tazobactam and 25% glycerol for 30 min and cryocooled in liquid nitrogen. Longer soaking times resulted in crystal deterioration to the point where no usable X-ray diffraction data could be collected, whereas shorter times resulted in an absence of electron density for bound inhibitor consistent with insufficient inhibitor diffusion/binding to give full occupancy throughout the crystal lattice.

CTX-M-15:PAS cocrystals were cryocooled by brief exposure (<5 s) to mother liquor supplemented with 5 mM inhibitor (to more closely preserve growth conditions and preserve crystal stability) and 25% glycerol within 24 h of crystal formation (note that there is no evidence in the electron densities of bound, intact inhibitor in the active site or elsewhere in the protein). Diffraction data for inhibitor soaks and cocrystals were collected at Diamond Light Source at beamline I03 (CTX-M-15:tazobactam-soak, apo-CTX-M-15^G238C^, and apo-CTX-M-15^G238/G239_Y240insA^), Soleil Proxima-2A (apo-CTX-M-15^E166Q^), Soleil Proxima-1 (CTX-M-15:tazobactam and CTX-M-15^E166Q^:tazobactam cocrystals), ALBA BL13-XALOC (CTX-M-15:enmetazobactam and CTX-M-15^E166Q^:enmetazobactam cocrystals), and DLS I04 (CTX-M-15^K73A^:enmetazobactam cocrystals and apo-CTX-M-15^K73A^). Data were integrated in DIALS (49) and scaled in Aimless in the CCP4 suite (50), with the exception of CTX-M-15^G238/G239_Y240insA^ apo data, which were integrated and scaled in autoproc (51). Phases were calculated by molecular replacement in Phaser (52) using PDB code 4HBT (34) as the starting model. Structures were completed with iterative rounds of manual model building using WinCoot (53) and refined using Phenix (54). Due to the ultrahigh resolution of the structures, hydrogens were added during refinement (refined using the “riding model” in Phenix) and all atoms (except hydrogen) were refined anisotropically. Occupancies were refined in Phenix over at least 10 rounds of refinement. Geometry restraints for the tazobactam-derived acylenzyme were calculated using eLBOW in Phenix. Figures were generated using PyMOL (55).

### Steady-state kinetics.

Nitrocefin hydrolysis was measured after 24 h for purified CTX-M-15^E166Q^, in the presence and absence of BLIs at a 1:1 ratio of enzyme to inhibitor. Reactions were followed at 482 nm using an Agilent 8453 diode array spectrophotometer (Agilent Technologies, Inc., Santa Clara, CA) in a quartz cuvette with a 1-cm path length. All reactions were conducted in phosphate-buffered saline at room temperature.

### Timed ESI-MS.

Homogenous preparations of CTX-M-15^E166Q^, CTX-M-15G238C, or CTX-M-15^G238C/G239_Y240insA^ were subjected to electrospray ionization-mass spectrometry (ESI-MS) after incubation with either tazobactam or enmetazobactam. A Synapt G2-Si high-resolution quadrupole time-of-flight mass spectrometer (Waters Corp., Milford, MA) equipped with a LockSpray dual electrospray ion source was used to acquire mass spectra. The instrument was calibrated with sodium iodide, using a mass range of 50 to 2,000 *m/z*; this calibration results in an error of ±5 atomic mass units (amu). All β-lactamase and β-lactamase–PAS reactions were terminated by addition of 1% acetonitrile and 0.1% formic acid in water, and samples were applied to an Acquity H class ultraperformance liquid chromatography (UPLC) instrument on a 1.7-μm 2.1-mm by 100-mm Acquity UPLC ethylene bridged hybrid C_18_ column (Waters) equilibrated with 0.1% formic acid in water. β-Lactamases and β-lactamase–BLI complexes were eluted using gradients with starting concentrations of 90% 0.1% formic acid in water (mobile phase A) and 10% 0.1% formic acid in acetonitrile (mobile phase B), reaching final conditions of 15% mobile phase A and 85% mobile phase B by 4 min. A gradient of 19% mobile phase A and 81% mobile phase B was reached by 1 min. The tune settings for each data run were as follows: capillary voltage, 3.5 kV; sampling cone, 35 V; source offset, 35 V; source temperature, 100°C; desolvation temperature, 500°C; cone gas, 100 L/h; desolvation gas, 800 L/h; and nebulizer gas, 6.0 Bar. Spectra were analyzed using MassLynx version 4.1 (Waters Corp.) and deconvoluted using the MaxEnt1 program.

### Tryptic digestion.

Five micrograms of CTX-M-15 or CTX-M-15^E166Q^ (incubated with enmetazobactam/tazobactam 1:1) was reduced (in 50 mM Tris [pH 7.5]) with 15 mM dithiothreitol (DTT; 37°C, 1 h) and alkylated with 25 mM IDA (dark, room temperature, 30 min). Proteolytic digestions were performed at 37°C with enzyme/substrate ratios of 1:25 by adding lysyl endopeptidase (Wako Chemicals) (30 min), followed by overnight trypsin digestion (Promega). Liquid chromatography-tandem mass spectrometry (LC-MS/MS) analyses were performed using an Orbitrap Eclipse mass spectrometer (Thermo Scientific) coupled with a nanoAcquity UPLC system (Waters). Proteolytic peptides (~300 ng) were desalted and concentrated with a C_18_ symmetry trap column (Waters) and separated using a C_18_ BEH130 reverse-phase column (Waters) with a linear gradient 0% to 42% mobile phase B (0.1% formic acid and acetonitrile) versus mobile phase A (100% water and 0.1% formic acid). Peptides were introduced into the nano-electrospray source at a capillary voltage of 2.0 kV. MS1 full mass spectra were acquired in the Orbitrap mass analyzer (resolution, 120 K). Tandem mass spectra were generated in the linear ion trap mass analyzer by collision-induced dissociation of peptide ions at a normalized collision energy of 35%. The resulting MS/MS spectra were searched against a CTX-M-15 protein database using Mass Matrix software with the mass errors of 10 ppm and 0.8 Da for MS1 and MS/MS scans, respectively. Methionine oxidation and cysteine carbamidomethylation were allowed for variable modifications, and the Ser70-Lys73 cross-link set as a mass shift of −18.010565 Da. Tandem mass spectra were further manually interpreted.

### Data availability.

Coordinates and structure factors have been deposited and are available at the Protein Data Bank under the following accession numbers: 6Z7J (CTX-M-15:enmetazobactam cross-link), 6Z7K (CTX-M-15:tazobactam soak/acylenzyme), 7BDS (CTX-M-15:tazobactam cross-link), 6Z7H (CTX-M-15^E166Q^:enmetazobactam cross-link), 7BDR (CTX-M-15^E166Q^:tazobactam cross-link), 7QQ5 (CTX-M-15^K73A^:enmetazobactam), 6Z7I (CTX-M-15^E166Q^), 7QQC (CTX-M-15^K73A^), 7R3R (CTX-M-15^G238C^), and 7R3Q (CTX-M-15^G238C/G239_Y240insA^).
