# Supplementary material for: Penicillanic Acid Sulfones Inactivate the Extended-Spectrum β-Lactamase CTX-M-15 through Formation of a Serine-Lysine Cross-Link: an Alternative Mechanism of β-Lactamase Inhibition
Source: mBio. 2022 May 25;13(3):e01793-21. doi: 10.1128/mbio.01793-21 (PMC9239225; doi:10.1128/mbio.01793-21)
Supplement: FIG S1 [file mbio.01793-21-s0001.pdf]

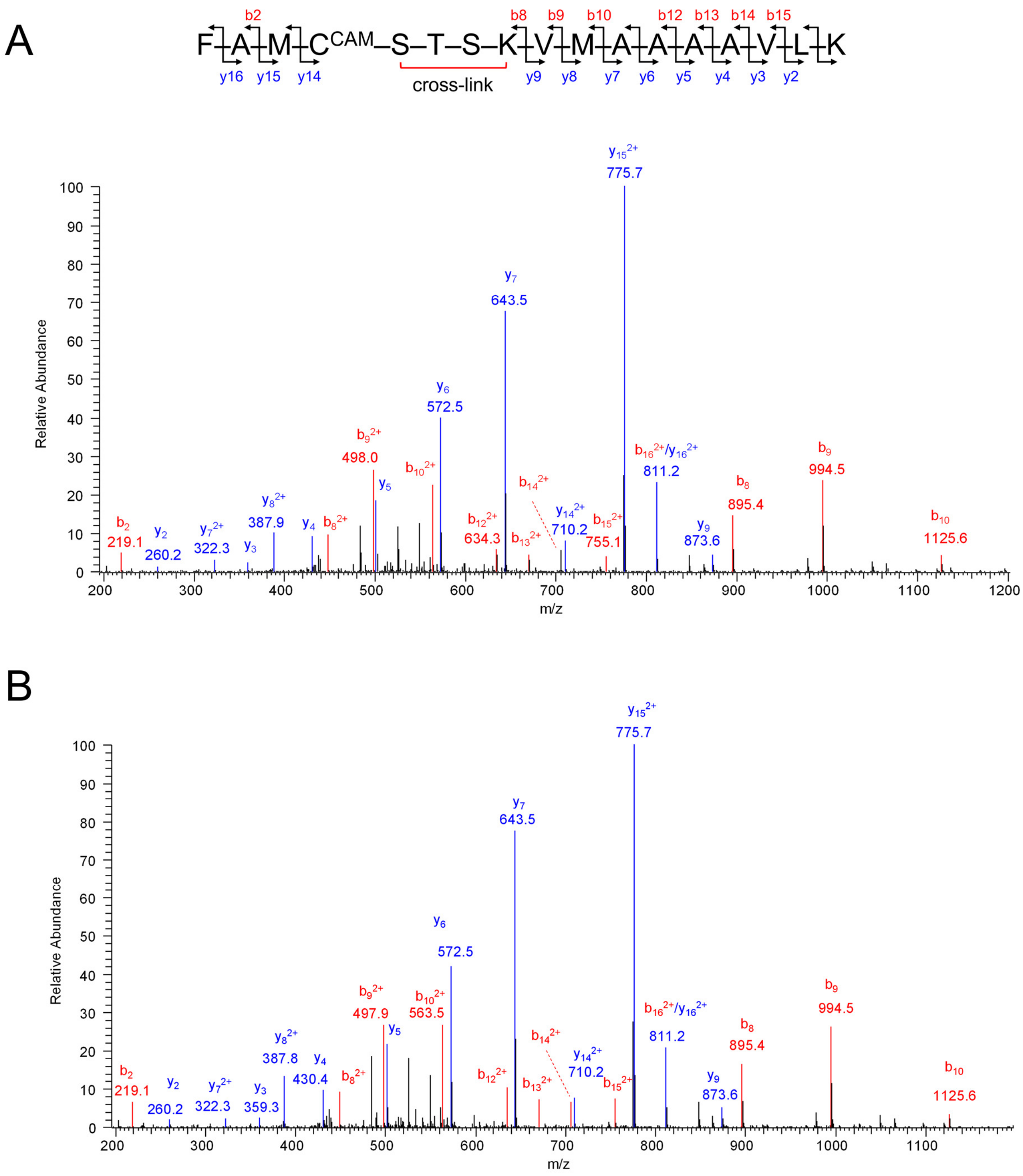

Figure S1. Tandem mass spectra of tryptic peptide FAMCSTSKVMAAAAVLK of CTX-M-15. Spectra of the FAMCSTSKVMAAAAVLK peptide (residues 66-82, with carbamidomethylated cysteine, C^{CAM}) after treatment with **(A)** tazobactam or **(B)** enmetazobactam. Several 'b' and 'y'-series ions were detected, but not between Ser70 and Lys73 (i.e. the cross-linked residues). A mass shift of -18 Da was observed at the precursor ion, y14, y15 and y16 ions, as well as b-series ions from b8 to b15, but not at b2 nor at y-series ions from y2 to y9, confirming formation of a crosslink between Ser70 and Lys73.
