# Supplementary material for: Penicillanic Acid Sulfones Inactivate the Extended-Spectrum β-Lactamase CTX-M-15 through Formation of a Serine-Lysine Cross-Link: an Alternative Mechanism of β-Lactamase Inhibition
Source: mBio. 2022 May 25;13(3):e01793-21. doi: 10.1128/mbio.01793-21 (PMC9239225; doi:10.1128/mbio.01793-21)
Supplement: FIG S2 [file mbio.01793-21-s0002.pdf]

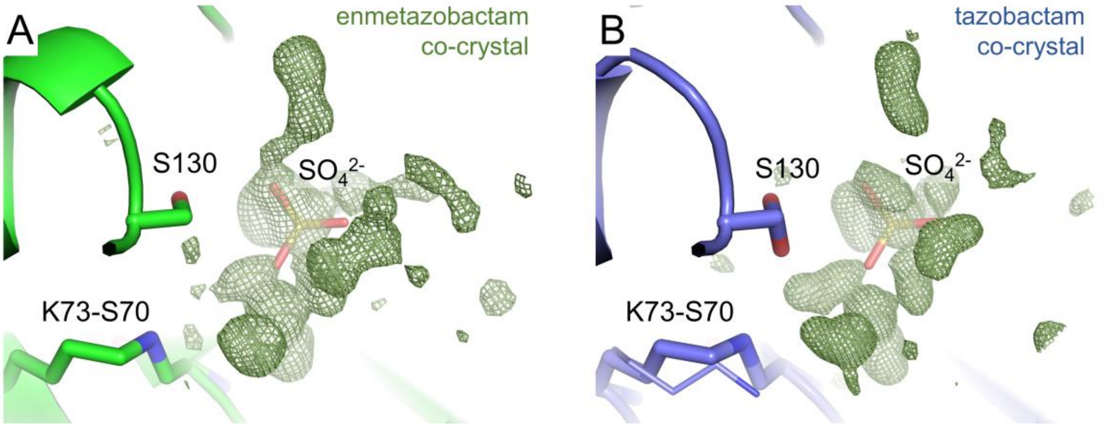

Figure S2. Residual positive *F*o-*F*c density in the active site of CTX-M-15 co-crystallized with enmetazobactam and with tazobactam. F_o-F_c density is contoured at 3σ and shown as a green mesh. The active site sulfate (labelled SO_4^{2-}) in native apo-CTX-M-15 [PDB 4HBT (34)] is overlaid for comparison. Views from the active sites of **(A)** CTX-M-15:enmetazobactam co-crystal (green) and **(B)** CTX-M-15:tazobactam co-crystal (blue).
