# Supplementary material for: Penicillanic Acid Sulfones Inactivate the Extended-Spectrum β-Lactamase CTX-M-15 through Formation of a Serine-Lysine Cross-Link: an Alternative Mechanism of β-Lactamase Inhibition
Source: mBio. 2022 May 25;13(3):e01793-21. doi: 10.1128/mbio.01793-21 (PMC9239225; doi:10.1128/mbio.01793-21)
Supplement: FIG S3 [file mbio.01793-21-s0003.pdf]

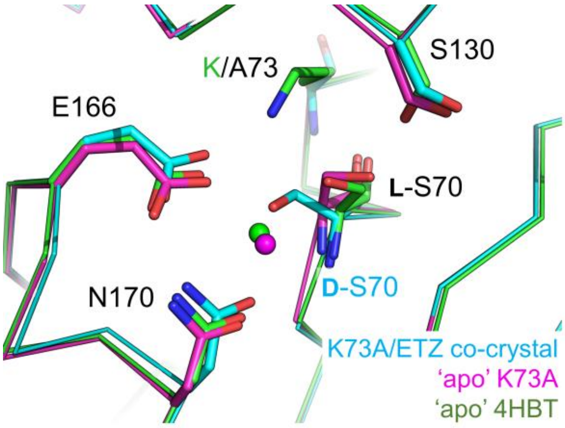

Figure S3. Comparisons of CTX-M-15K73A and CTX-M-15 native structures. Views from the active sites of CTX-M-15^{K73A}:enmentazobactam (ETZ) co-crystal (cyan), apo-CTX-M-15^{K73A} (pink) and native apo-CTX-M-15 [4HBT, green, (34)].
