# Supplementary material for: Penicillanic Acid Sulfones Inactivate the Extended-Spectrum β-Lactamase CTX-M-15 through Formation of a Serine-Lysine Cross-Link: an Alternative Mechanism of β-Lactamase Inhibition
Source: mBio. 2022 May 25;13(3):e01793-21. doi: 10.1128/mbio.01793-21 (PMC9239225; doi:10.1128/mbio.01793-21)
Supplement: FIG S4 [file mbio.01793-21-s0004.pdf]

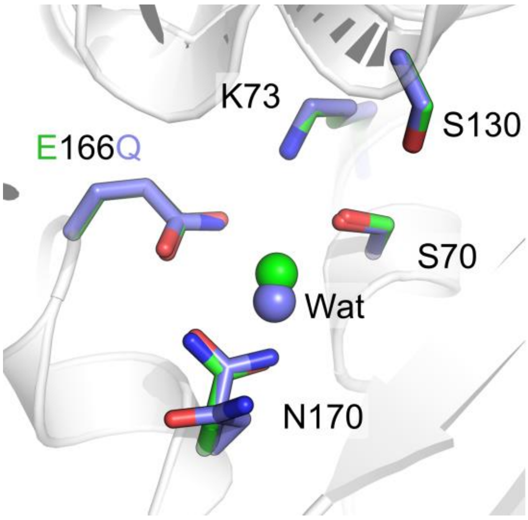

Figure S4. Conformation of CTX-M-15^{E166Q} active site residues. Superposition of unliganded CTX-M-15^{E166Q} (blue) with native, unliganded CTX-M-15 [green, PDB 4HBT, (34)]. Asn170 is in two conformations, with the major conformation in CTX-M-15^{E166Q} (occupancy 0.78) oriented differently compared to 4HBT. The catalytic water (Wat, green or blue sphere) moves 0.8 Å due to the movement of Asn170. Mutation of Glu166 to Gln does not affect backbone or side-chain geometry.
