# Supplementary material for: Penicillanic Acid Sulfones Inactivate the Extended-Spectrum β-Lactamase CTX-M-15 through Formation of a Serine-Lysine Cross-Link: an Alternative Mechanism of β-Lactamase Inhibition
Source: mBio. 2022 May 25;13(3):e01793-21. doi: 10.1128/mbio.01793-21 (PMC9239225; doi:10.1128/mbio.01793-21)
Supplement: FIG S5 [file mbio.01793-21-s0005.pdf]

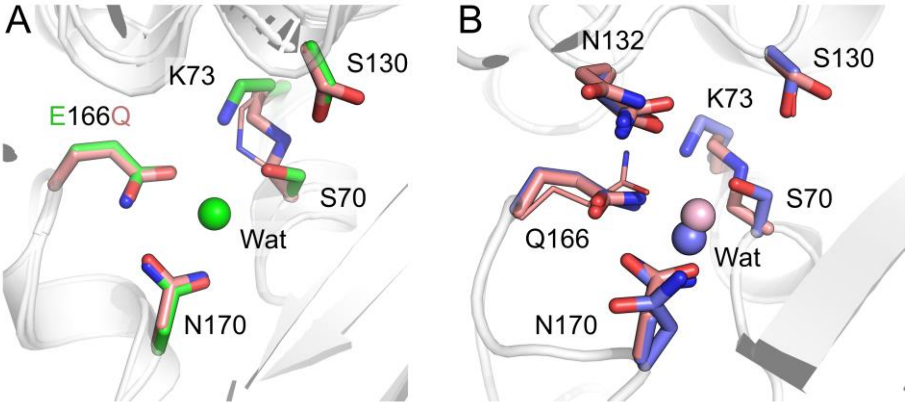

Figure S5. Movements of active site residues in CTX-M-15^{E166Q}:PAS co-crystal structures. (A) Superposition of CTX-M-15^{E166Q}:enmetazobactam (pink) with native, unliganded, CTX-M-15 [green, PDB 4HBT (34)]. Asn170 adopts one conformation, identical to that in 4HBT. However, there is no electron density for the catalytic water in CTX-M-15^{E166Q}:enmetazobactam. **(B)** Superposition of CTX-M-15^{E166Q}:tazobactam (pink) co-crystal structure with unliganded CTX-M-15^{E166Q} (blue).
