# Supplementary material for: Penicillanic Acid Sulfones Inactivate the Extended-Spectrum β-Lactamase CTX-M-15 through Formation of a Serine-Lysine Cross-Link: an Alternative Mechanism of β-Lactamase Inhibition
Source: mBio. 2022 May 25;13(3):e01793-21. doi: 10.1128/mbio.01793-21 (PMC9239225; doi:10.1128/mbio.01793-21)
Supplement: FIG S6 [file mbio.01793-21-s0006.pdf]

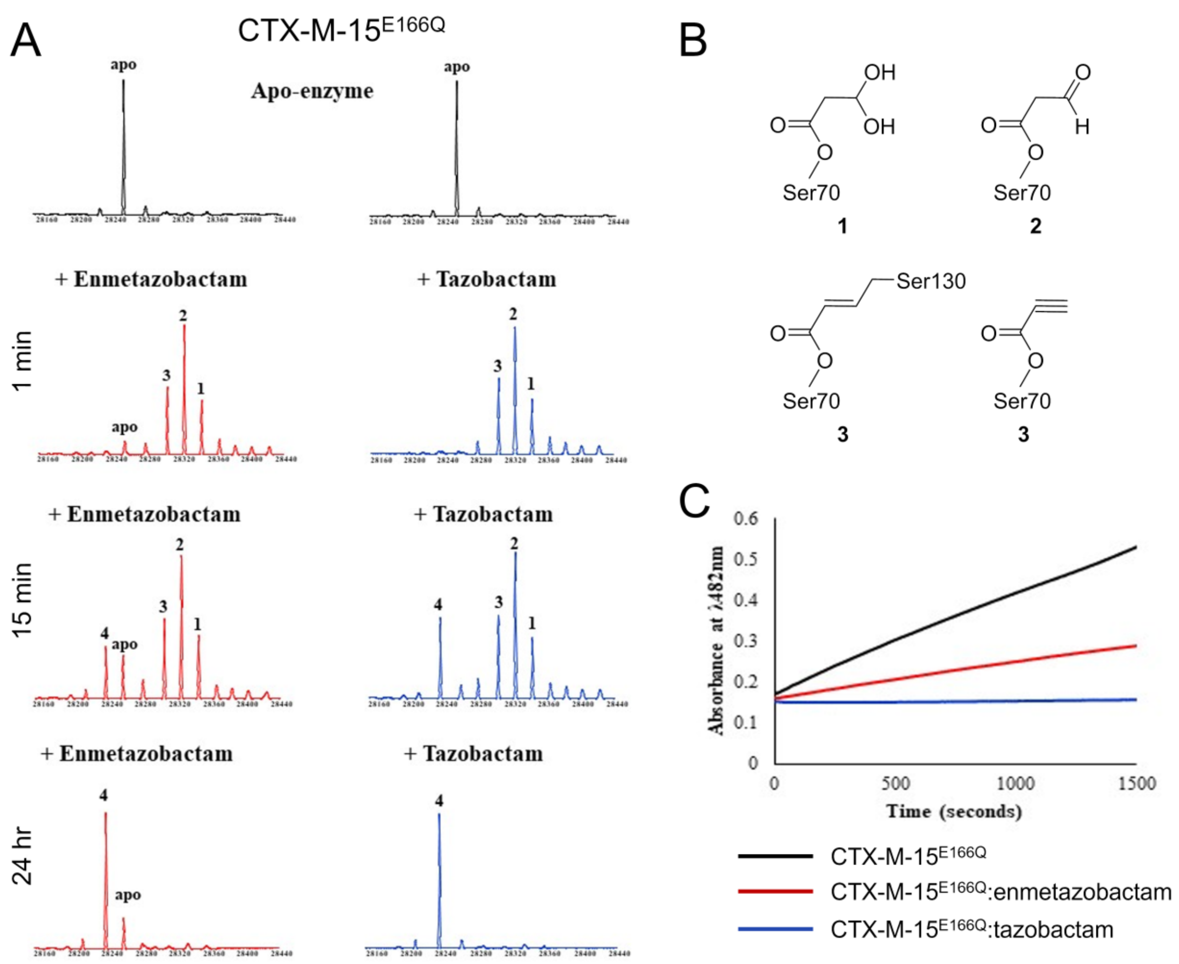

Figure S6. Intermediates formed on reaction of PAS compounds with CTX-M-15E166Q revealed by timed electrospray ionization-mass spectrometry (ESI-MS). (A) Mass spectra showing the formation of intermediates during inactivation of CTX-M-15^{E166Q} (apo, 28257 Da) by enmetazobactam or tazobactam at 1 min, 15 min and 24 hour incubations. Previous work conducted by Papp-Wallace et al., [ref (21), Figure 4] showed the ESI-MS of apo-CTX-M-15 and complexed with enmetazobactam and tazobactam. Analyzing ESI-MS of CTX-M-15^{E166Q}, the deacylation deficient variant, peaks 1 (+ 90 Da), 2 (+ 69 Da), and 3 (+ 50 Da) correspond to post acylation fragmentation of the two PAS compounds from their enamine/imine intermediates [**Figure 6**, see also (21)]. Peak 4 is the –18 Da modified apoenzyme (containing a Ser70-Lys73 cross-link, **Figure 7**). **(B)** Possible breakdown products corresponding to peaks 1, 2 and 3 in A, as identified previously (21). Two breakdown products of equivalent mass, including the Ser70-Ser130 cross-linked species (21, 28, 29) may be represented by peak 3. ~50 Da adducts represented by peak 3 have also been shown to form on reaction of PAS compounds with both GES-2 (34) and KPC-2 (21) and can break down to yield active enzyme (KPC-2) or, as here, lead ultimately to formation of the catalytically inactive Ser70-Lys73 cross-linked species. **(C)** Nitrocefin hydrolysis by CTX-M-15^{E166Q} after 24 h incubation with enmetazobactam (red) or tazobactam (blue). The observed hydrolysis by the CTX-M-15^{E166Q}:enmetazobactam protein after 24 h incubation is due to the small amount of unmodified enzyme present in this mixture (see mass spectrum at 24-h timepoint, panel A bottom left).
