# Supplementary material for: Penicillanic Acid Sulfones Inactivate the Extended-Spectrum β-Lactamase CTX-M-15 through Formation of a Serine-Lysine Cross-Link: an Alternative Mechanism of β-Lactamase Inhibition
Source: mBio. 2022 May 25;13(3):e01793-21. doi: 10.1128/mbio.01793-21 (PMC9239225; doi:10.1128/mbio.01793-21)
Supplement: FIG S8 [file mbio.01793-21-s0008.pdf]

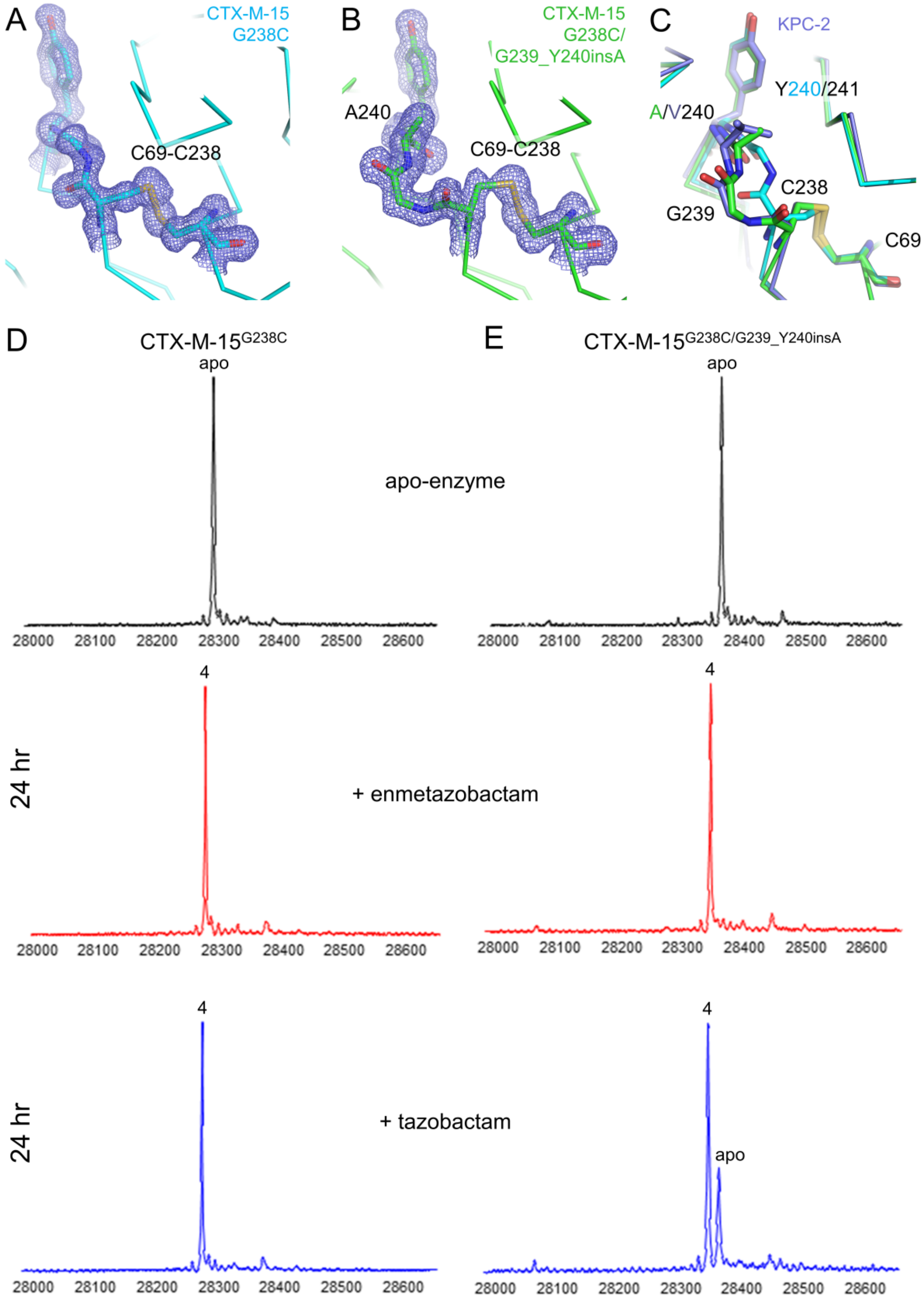

Figure S8. Effect of a C69-C238 disulfide bridge on cross-link formation in CTX-M-15. Disulfide formation in the CTX-M-15 mutants defined by 2F_o-F_c electron density (blue mesh, contoured at 1σ): **(A)** CTX-M-15^{G238C} and **(B)** CTX-M-15$^{G238C/G239_Y240insA}$. **(C)** Overlay of CTX-M-15^{G238C} (cyan), CTX-M-15$^{G238C/G239_Y240insA}$ (green) and KPC-2 [PDB 5UL8 (56), magenta] active sites. Note that the CTX-M-15$^{G238C/G239_Y240insA}$ loop is structurally similar to the equivalent loop in the KPC-2 carbapenemase, in which cross-link formation following PAS exposure is not observed (21). **(D)** and **(E)** Mass spectra, *below*, showing the formation of intermediates during inactivation of CTX-M-15 mutants by enmetazobactam or tazobactam after 24-hour incubation for **(D)** CTX-M-15^{G238C} (apo, 28306 Da) and **(E)** CTX-M-15$^{G238C/G239_Y240insA}$ (apo, 28377 Da). Peak 4 is the -18 Da modified apo enzyme representing formation of the Ser70-Lys73 cross-link.
