# Supplementary material for: Penicillanic Acid Sulfones Inactivate the Extended-Spectrum β-Lactamase CTX-M-15 through Formation of a Serine-Lysine Cross-Link: an Alternative Mechanism of β-Lactamase Inhibition
Source: mBio. 2022 May 25;13(3):e01793-21. doi: 10.1128/mbio.01793-21 (PMC9239225; doi:10.1128/mbio.01793-21)
Supplement: FIG S9 [file mbio.01793-21-s0009.pdf]

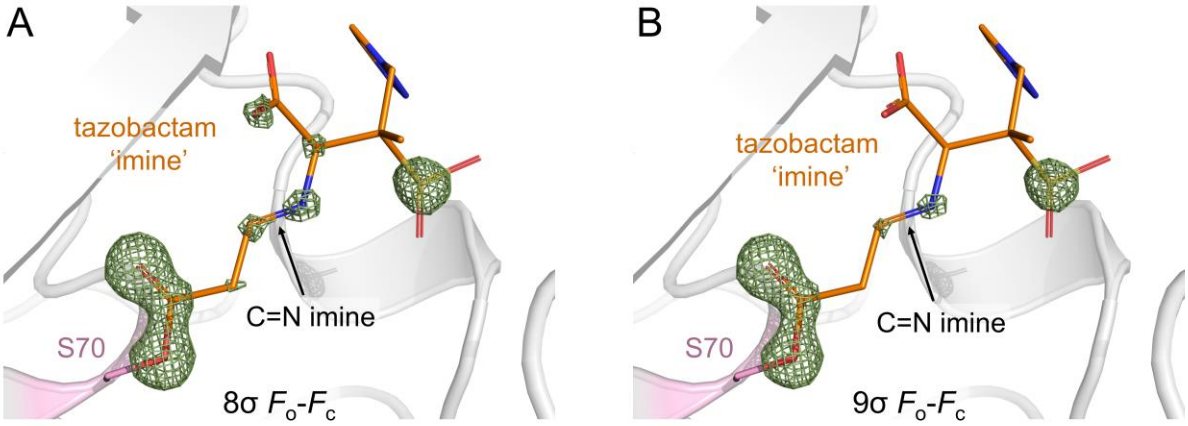

Figure S9. Omit electron density for tazobactam bound to CTX-M-15. F_o-F_c density calculated in the absence of ligand is contoured at **(A)** 8σ or **(B)** 9σ. At this resolution (1.1 Å), the C=N bond length observed in the crystal structure (1.29 Å) is shorter than the expected values (Allen FH, Kennard O, Watson DG, Brammer L, Orpen AG, Taylor R, Perkin transactions 2:S1-S19, 1987, https://doi.org/10.1039/P298700000S1) for a C-N or C-C bond (1.47 Å/1.50 Å, respectively).
