# Supplementary material for: Penicillanic Acid Sulfones Inactivate the Extended-Spectrum β-Lactamase CTX-M-15 through Formation of a Serine-Lysine Cross-Link: an Alternative Mechanism of β-Lactamase Inhibition
Source: mBio. 2022 May 25;13(3):e01793-21. doi: 10.1128/mbio.01793-21 (PMC9239225; doi:10.1128/mbio.01793-21)
Supplement: FIG S10 [file mbio.01793-21-s0010.pdf]

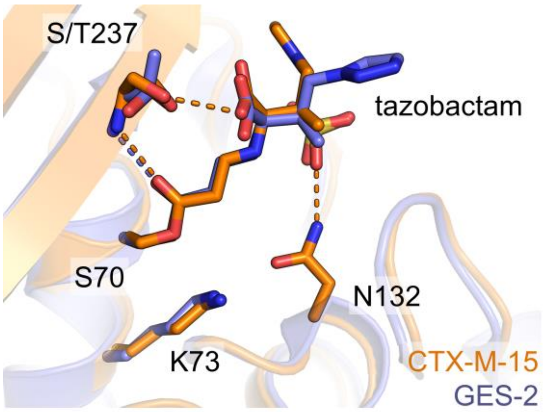

Figure S10. Comparison of tazobactam binding to GES-2 and CTX-M-15. Superposition of CTX-M-15:tazobactam (orange, 1.1 Å resolution) with GES-2:tazobactam [blue, PDB 3NIA (30), 1.65 Å resolution]. Interactions are shown as colored dashes corresponding to the protein color. In GES-2:tazobactam the ring-opened form of tazobactam interacts only with the backbone amide of Ser237.
